# Supplementary material for: Feedback of individual genetic and genomics research results: A qualitative study involving grassroots communities in Uganda
Source: PLoS One. 2022 Nov 18;17(11):e0267375. doi: 10.1371/journal.pone.0267375 (PMC9674126; doi:10.1371/journal.pone.0267375)
Supplement: S1 File — (DOCX) [file pone.0267375.s001.docx]

**FOCUS GROUP DISCUSSION 006 GRASSROOTS COMMUNITIES ABOVE 35 YEARS, EASTERN**

**We would like to discuss with you a subject that can directly or indirectly affect your lives and your health. In order to guide our discussion, we shall ask you some questions.**

**Awareness/understanding of genes and genome**

**Qn1. What can we possibly inherit from our parents? *(physical, behavioral, health conditions)***

*R3: There are diseases that a child can inherit from the father or the mother like sickle cell and also high blood pressure.*

R4: (Interjection): Also cancer!

*R2: But I do not think cancer is hereditary, that now depends on the type of cancer but the transmission of cancer depends on an individual. Those diseases that can be inherited are there but those are the two that I know.*

*R5: Asthma, syphilis and HIV virus are also transmitted through blood. If the father or mother has the disease of the bones, the child will inherit it also.*

*R7: Thank you! There are things that we inherit from our parents, especially diseases. When a pregnant woman and her husband go to hospital for antenatal care, they normally ask both of them “do you have epilepsy or not?” if they say “no”, they will indicate “no” on the medical form. Whether you have sickle cell or any other thing that the unborn child can inherit, all that will be captured during antenatal time. But another thing that we can inherit from our parents is the skin colour or facial appearance. If the mother is brown, the child will also inherit that. If the mother or father is dark skinned, that child will also be dark skinned. Thank you****!***

*R3: On my side, the diseases that can be inherited include sickle cell, tuberculosis, HIV, candida and syphilis.*

*R8: I don’t know whether people have understood that question very well. There is a way God has created us. There are things that God has created in us that can be traced to the grand fathers and that have been passed on to us through blood. It is like syphilis, this is minor. There are the other ones that we are created with*

*R4: (Interjection): Like epilepsy!*

*R1: Exactly! Like that one, these are the ones we are discussing and also asthma. Then I was also wondering about the structural appearance of people. There is a way they have created people like for us the Itesots we are different from the way Baganda are. So I think structurally as well.*

*R5: I can also inherit height from my parents. For example if my parents are short, you don’t expect me to be tall, they will enventually conclude that I am a bastard or my mother cheated on her husband. So I can inherit height the parent’s height, the parent’s colour and then the parent’s character. If my father is abusive, I am also likely to be abusive and if my mother is quarrelsome, eventually I will be quarrelsome.*

*R3: The child inherits the appearance from the mother. If the mother is dark skinned, the child will also be dark skinned. If the father is light skinned, the child will also be light skinned and if the mother has bad manners like practicing witchcraft the child will also inherit such bad manners. The child inherits the character of the mother or the father.*

*R6: On tribal grounds, I can easily tell that this is an Itesot, this one is a northerner, this one is a westerner because God just put us there and when you look at our whatever (appearance), you can easily tell by hair because Itesots, I normally identify them by the type of hair, very easy to tell. And when you go to the west, you can easily tell by the nose and how they speak (accent).*

**Mod: Thank you!**

**Qn 2: Which health conditions can one inherit from biological parents?**

R8: I think they are taking us back (to the previous question).

**Qn 3: What do you understand by the word gene? What about DNA?(Moderator explains)**

R6: Those are the same things that are transferred (from the parents’ to the child).

R3: Those are things that a child inherits from the parents. That thing might have started with your father. That is why you find that a child inherits the physical appearance also. But there are things associated with good life like if the father is fat, the child will also grow fat. You find that the children are also fat. Those are the things that are inherited and also character. You find that the children inherit character. When you produce your children, there are those ones who will inherit your character. Much as the parents are two, the child will genetically lean more on one parent.

**Mod: (Explains the meaning of a gene).**

**Qn 4. What about a genome?**

R5: Let me say (something) in relation to the DNA. If I see that this child resembles another person then it means that this is not my blood. The people who produced that child are different from me. That means they are going to test the blood of that child and my blood in order to confirm whether the child is mine or not. It means that there are things that God created in me that are unique from what other people have and I can tell that that this child is mine or not.

R7: The DNA is what distinguishes one person from another. For example you can tell that this child does not belong to this family and does not belong to the other family. Because of the way things are, if this family is dark skinned and a child happens to be light skinned, it is considered a curse or associated with cheating (by one of the couple) . This child does not belong to that family. It means that, that child should not stay with that family and the mother should take that child where it rightfully belongs.

**Mod: (Explains the meaning of DNA)- They are saying that, it is something unique in relation to the nature of a humanbeing or an animal. That is why you find that if it is a fish, that thing will grow into a fish, if it is a cat, that thing will grow into a cat. It is something related to the skin of the child.**

.**Explanations**

**Thanks for your contributions. Let us share more about these two issues. Each species or group of organisms has a unique set of inherited characteristics that make them different from each other e.g. color of skin, eyes, height. These characteristics are usually encoded/incorporated in the DNA molecules present in their cells.**

**DNA is the genetic building blocks that govern all the characteristics of a living thing (e.g. animals, plants, and bacteria).**

**Genes are the elements of heredity that determine what is transmitted from parents to offspring in reproduction. Genes are composed of specific DNA portions or segments that are capable of controlling specific heredity characteristics.**

**Genome is an organism’s complete set of DNA, including all of its genes. Each genome contains all of the information needed to build and maintain that organism. Genome is the entire quantity of heredity information. It can also be applied to refer to specific genetic contents which may also comprise of non-chromosomal genetic elements such as viruses, plasmids and transposable elements.**

**An organism has one genome, but it has thousands of millions of genes in that particular organism.**

**Your genetic information can be similar to that of your immediate family, and blood relatives. Your decisions concerning testing and what to do with the results may affect them as well.**

**Health professionals are ethically responsible to prevent harm to those involved.The individual tested also has responsibilities and obligations and should appreciate the shared nature of genetic information within families.**

**In some cases, genetic tests provide reliable and accurate information based on which decisions could be made e.g. … while in some cases, it is not possible to get definitive results. Prediction of a potential condition or disease may not include the severity of the condition or the age for the onset of symptoms. An individual is much more than the sum of their genes: the individual’s environment can modify the expression of genetic messages to the body. Many health factors are not genetic.**

# Attitudes towards applications of modern genetics and genomics

We are going to discuss specific issues to do with genetics and genomics. We would like to hear your views from the stand point of your ethnic group or culture and from your religious back ground. You could also tell us what you personally think.

## Qn 5. Genetic testing

A genetic test examines your DNA, and can reveal changes or variations in your genes that may be associated with an illness or a disorder. A genetic test can be arranged by your doctor or health clinic.

- 1. **Who among us has experience with taking a genetic test? (Could be you or someone close to you). What was your experience with the process of getting the test? (Prompts: informed consent …information on potential ethical issues etc.)**

R1: DNA testing- In Teso if a person has defiled a child and he is denying responsibility of the pregnancy, they will take him and the girl for testing in order to determine the blood group of each of them. They will also check the pressure of the girl and the man. Then they will test for diseases (inherited). If they find genetically inherited diseases that you have like diabetes and sickle cell in the blood sample of the child, they will conclude that you are the one who impregnanted the girl. They also test the sperm of the man to confirm that it fertilised the ovum of the woman. The machine will show that it is this man’s sperm that ferterlised the girl’s ovum and they will conclude that he is the one who impregnanted the girl even if he is denying

R6: Let me say a DNA test is done to identify someone especially in our culture of Teso, it is common for a woman to cheat on the husband and for the man to cheat on the wife. You find that the man has impregnanted another woman outside marriage but when they say that he is the one responsible, he will not accept so they normally conduct a DNA test. They will wait until the woman has delivered and then they take the blood sample of the woman, the man and the child for testing. This DNA test is done in Entebbe but we have never witnessed its procedure. We only hear that they will take the blood samples of the man and the child for testing in order toacertain whether the man is the actual father or not. In case of sickle cell, we might look healthy yet we have the gene in us and we are carriers. When you get married and both of you are sickle cell carriers, you will produce children with sickle cell. Even if it is only the man who is a carrier, you will still produce children who are carriers. So this DNA test is done and when it is done it will confirm that your child has sickle cell. Sickle cell can not be hereditary if both parents do not have that gene. You must be having that gene for the child to inherit sickle cell from you.

R4: I also want to add, this has also ever happened in our clan. There was a scenario when the parenthood of the child was in doubt. Just like what my sister said (previous speaker), most married couples cheat on each other and when a child is born resembling another person, the man concludes that the child is not his. I heard that they went and tested blood and it was confirmed that the child was not for the man. The father to this child was different. This testing comes with advantages and disadvantages to family. That is what I know. But the man was very happy to comfirm the truth because they used to allege that the child was his.

R7: I want to acknowledge that my father made my sister to go through the same. At first he denied being the father to my sister but when they went for a DNA test, it was confirmed that he was the true father. He no longer has any doubts and he is instead happy now.

- 1. **At some point in life, one might consider having a genetic test to find out their risk of developing an illness or a disorder. What do you feel about making genetic tests available so that people with a family history of serious genetic diseases can find out if they are at risk?**

R6: Let me say that this testing is very good only that it might not be accessible but it is very good to test because it helps us to discover what runs in the blood line of the family. During our time when we were marrying, due diligence was done to find out the diseases that run in the blood line of the family you are marrying from but now days young people get married anyhow. That is why you find that they get married to someone with a genetic disease and they end up suffering. If it were possible, it would be necessary to carry out genetic testing in all families to discover the genetic diseases they have. It is very good to test because we get to know that such and such a family has these particular diseases. It would give people even better life and better development because some people are hindered even in developing because when you marry someone who is a carrier of sickle cell, now you will suffer treating this person and yet you did not know that person/family has sickle cell. So if there was an easy way of even checking most of the families it would save, it would really save them.

R1: I would really appreciate it for several reasons. One, it will save the family that is going to marry from a particular family and even the children and at the same time also help the people having that problem in the sense that they will be able to know earlier so that they know how to take care of such people. So it saves a great deal. The problem is, it is abit expensive and then those services are very far, otherwise I would really say that it is a good thing to do.

R8: Sickle cell disease check up is now a routine and we appreciate the idea. It is now a routine for pregnant women. During antenatal care, they do tests to prevent it because now when you produce a baby with sickle cell it disturbs a lot. Then may be the other one where people find it very difficult is about the DNA, like when you marry a man whose rhesus factor is positive and may be you are negative, but for that one it is very difficult to test. We have never got access to that.

R5: That test is very good. It is after testing that you get to know that the other person is suffering from cancer of the bones or tuberculosis or they have various diseases. It is through these tests that various diseases are discovered from somebody.

R3: It is good to test because it benefits the family, in other words all families live peacefully. It will save families from wasting money. It also helps patients to find ways of treating themselves.

R2: It will help those who are married to live with precaution. It will help us to be conscious of our health.

R6: It is good to test because it helps the family to live peacefully without any conflict.

R7: If a student has undergone that DNA test on other diseases, the school can have the history through the medical report from the doctor or from the family, so the parent will eventually be able to introduce the child to the school and the school will probably notify the rest that “don’t mistreat this one or don’t beat him or her”. So special attention will be given to that particular child and will find education very simple and will perform well academically

R1: When we were still young, we never got a chance to go through genetic testing. The world has changed. People these days go for genetic testing because these diseases might attack the family. You might think that your child has been bewitched or he/she is suffering from HIV and yet the child is suffering from cancer of the bones. Testing brings peace of mind.

**Qn 6.0. Return of results**

**6.1. What would make you comfortable and confident to participate in GG research?(probe on issues of confidentiality, sharing of results with other members of the family or blood relatives,… use of materials for other studies other than the current, transfer of materials outside the country etc.)**

R5: What I think is that there should be a projector to show us photographic images of this genetic science and the procedure of genetic testing. When you see that the other child resembles the parents it makes you to appreciate that you are studying something that exists.

R4: To bridge the gap of language barrier like when somebody is an illiterate and does not know how to read and write, if the research department was able to bring the projection in form of a video, somebody who does not know how to read will be able to interprete what is going on. So it would influence that person and attract more attention on that. Thank you

R6: May be improving on learning resources or materials to make people understand things better.

R8: Somebody (a colleague) is saying the challenge is that people have studied English upto primary and they are not getting their mother tongue, so this approach of writing in English and alsoAteso is a very good approach. We appreciate the fact that you have used both languages because not everybody in Teso knows Ateso, maybe they just know how to verbally speak, but when it comes to reading what is written they cannot.

R7: Availing information out there to the people explaining the importance of doing it and why we do it can really play a very big role in causing (a positive) change in attitude and perception of the people. So that is what I would suggest that we continue doing. It will be good to reached out to more people.

R1: Then other one can be some bit of “retreat” as far as time is concerned. If the deparment was able to come up with various questions on the same topic, then it would be scheduled in form of a “workshop” for a day, two days or so, it would make people interact very well.

**6.2. If there was a study on GG that could potentially reveal that you/ your family or communities are susceptible to certain diseases which are very difficult to treat, would you consider participating? Would you want the research results if you participated? Would you be willing to share the results with immediate family, blood relative, community and beyond?**

R2: I do accept it especially if the person is from my clan or family. I do accept my family members to be tested.

R7: I accept it. They should continue to sensitise people because there are many genetic diseases that people are ignorant about. Sensitisation helps people to know the genetic diseases that run in their blood line. For example, for us in our family we know that high blood pressure runs in our blood line, so if one of the family members develops it, it will not be a surprise.

**Mod: How about sharing results with other people in the village?**

**Mod: Your results because they have finished to test you.**

R5: Sharing my results or their results?

R7: It is good to share results with other people because it safeguards their health. If a person knows that I have a particular genetic disease, it will be up to them to decide whether to produce with me (children) or not. If a person chooses to marry me, that is their risk.

**6.3. If you chose to take part in genetics testing, what would make you comfortable and confident to participate in heredity testing? (probe on issues of confidentiality, sharing of results with other members of the family or blood relatives,… use of materials for other studies other than the current, transfer of materials outside the country etc.)**

R4: I think it is basically the knowledge after getting the information that really prepares you to be free. Now like us at least we have heard and we have gotten to know what it is all about, so it gives me the freedom (courage) to participate freely without the fear that I had before

R1: When I receive feedback at the right time and there are no other discouragements and at the same time the person who is giving me feedback first begins by counselling and guiding me, reminding me of what went on and how to live afterwards.

R3: Therefore the counselling that is given before the report that has been brought from there(research center) can excite and encourage me to continue to do that test even to my clan members.

**6.4. If there was testing on heredity that could potentially reveal that you/ your family or community are suffering or susceptible to certain diseases, would you consider participating? Would you want to know the testing results if you participated? Why? How would you like the results to be communicated?**

R7: To inform each person individually and avoid availing them information in a group because by so doing you’ll end up killing some people (he then laughs).

R1: What method do you want to be used to be given that information?

R5: Each person has to be given results individually. If it is like a family and it is one at a time and they are the ones to take back to the rest.

**Mod: Any other person?**

R3: I accept to be given the results alone when they come back.

**Mod: Do you accept to participate in that screening?**

R2: Testing is good and people cannot reject, even in the family when you test and know what you are suffering from you can easily plan. It also eases work for the researchers to know exactly what disease one is suffering from. The way results are disclosed, if it’s me the doctor can disclose to only me, if it’s the family the doctor can disclose to the whole family as we all hear so that everyone knows what disease is spreading in the family. It’s not something that can be kept secret, it needs to be clear so that even my other brother knows that in future when I grow older I might develop that disease.

6.5. **Should results of heredity testing be shared? With who? Patient? Family? Community? How should they be shared?**

**Why would results of heredity testing be shared?**

R8: Because it helps on the side of treatment and health and unity of the family.

**Mod: Why is it necessary to share this information?**

R1: It helps the family to understand the problem that they are faced with so that it’s able to plan together, how to help in case there is any one sick and others are not, the family can understand how to plan and handle such situations.

**6.6 How should results of heredity testing be shared? Would you be willing to share the results with immediate family, blood relative, community and beyond?**

R6: We accept.

R7: I need to share with my family so that even the family of my sister is also aware that my brother’s family is suffering from such and such a disease, it possibly may not be in my sister’s family. But I can not again cross and share with the other family

**6.7. Following genetics testing, sometimes findings rather than those intended are discovered. Should such incidental findings be shared? How should they be shared? With who? For what reasons?**

R6: It is fine to inform me that you have taken a sample of my blood and it has been tested for my gene but there is a certain disease like this, such that I get to know before my family.

Mod: How do you want this information shared with you? Should you be given the results and you read for your self or they should they come and share with you?

R2: If am given the results that read, I don’t know how to interprete, they are the ones surposed to inform me that they have tested my blood sample and found a certain disease. And I also get contented, then you counsel me and I get to know the truth, may be it’s a terrible disease like HIV, and I request them to call even my family and you help explain to me while my family is also present.

R3: It’s necessary for you to be tested so that you personally know your health status. And now the issue of sharing with my family when results come back to me, I am the one to decide to share it with my family.

R8: Especially the issue of telling some body that you have another disease apart from the ones you have tested for, you again have to repeat couselling me and my people. Because now as we talk, there is a machine that has reached us in Serere, “ that machine, you will only pay 10,000 shillings, after you have put the 10,000 shillings on the table, they will tell you ‘move to the machine’. So as you move to the machine, the machine runs for a short time and then tells you”,you have twenty three diseases Then somebody is heart broken, gets confused or you went with the aim of may be knowing what is paining my stomach, then all of a sudden you are made to stand next to the machine and the machine speaks in English that “you have 23 diseases” and you pay this amount of money and it is a big amount. So sometimes the way you also break the information to somebody matters.

**6.8. Results of hereditary testing of an individual usually reveal the genetic information of other family members and or community. What are the implications of sharing heredity testing results? Should such sharing be done? How can it be done safely?**

R7: As we said earlier that it’s necessary to share, but using the right way of approach to explain to me. And also what kind of approach are you going to use to explain to me and my family such that the family and I too remain at ease. So the approach matters-how you are going to break for me the news, how you are going to give me the report about my result matters.

**Mod: Is there any other person?**

R4: So you tested me, come back, give me my results after counselling me because some results may be scaring. Somebody needs to be prepared, come and prepare me, talk to me, counsel me and then see how you can (break the news). If it is a couple then you need to prepare the man, if it is a woman, prepare the man also before you break the news then of course see how to go with it. If it is a family then you go on. There are stages-individual, if it is a couple then you go to the family, then you go to the extended family, the clan depending. You have to be careful who should get those results (maintenance of confidentiality) because even in families today people hate each other, otherwise it may end up becoming a real issue again in the family that has to be solved. Those are the channels: individual, couple and then family. How (procedure) by siiting one down and explaining the situation that this is how it is, that is what I think.

**6.9. In view of what we have discussed, what other concerns do you have, if any, aboutheredity testing?**

R3: There is no bad information as yet.

R6: It’s concerning testing, it should now be like a bi-law in clans for testing because in families that got married long ago and were not tested there are alot of problems so far. Because some got married without knowing those diseases where by most of them are facing challenges in their marriages because of these gene issues and they don’t know what it is about. Therefore I encourage the government to make testing mandatory when a child grows up at that stage, they should be tested, the genes should be tested. With a card that is given to the child to show the gene such that when the time reaches to marry the parents can ask the man and woman their cards so that the parents have the awareness to reduse conflicts in families.

R2: My last concern is that just as she was explaining, after testing, I don’t know how much research is doing in your deparment such that incase this problem has been identified in me, there must be some treatment, otherwise if you leave me the way I am after realising this heavy thing, then you just leave me without any hope, I think it will discourage so many people. So as much as you are doing this by testing the genes there should be some development on the other side to counter the problems that we meet. To me that would be a very big blessing.

**Mod: Is there any other needed to be added**

(Silence)

**Mod: Thank you very much for your ideas (A respondent interrupts and cuts short the closing remarks).**

**Thank you for your contributions!**

**FOCUS GROUP DISCUSSION 007 GRASSROOTS COMMUNITIES YOUTH, NORTHERN**

**M:** **Question number one says what can people inherit from our parents? Are we listening? What can we as children inherit from our parents. That is our first question. So if anyone has an answer please raise up your hand so that we bring the microphone to you so that while you are answering we can record your voice. What can we inherit from both our parents because when 2 two of them come together they form a person, right? What can we inherit?**

R3: We can get HIV/AIDS.

R5: Sickle cells.

R2: Colour

**M:** **Skin colour?**

**M:** **Any other response?**

R8: In my own opinion a child can inherit how their parents behave. If the father is active and maybe the mother is dormant, the child can inherit the active side of the father, the child cannot inherit both the active and dormant part of both parents all together.

**M:** **Any other response? If we don’t have any other response, let’s go to the next question. Question two says; what medical conditions can we inherit from our parents? What medical conditions can a child inherit from their parents? Let’s think in terms of diseases that a parent can pass to a child especially genetic issues. Are we listening? Anyone who has an answer can please respond to this question**

R10:  We can get HIV/AIDS

R8: We can get diseases like Sickle Cells.

**M:** **Any another response? We can get sickle cells, we can also get HIV/AIDS.**

R7: We can get diseases like AIDS the Virus.

**M:** **I request that you speak a little bit louder because we need to capture the voice otherwise we will not get what you have said**

R5: We can get diseases like hepatitis.

R9: First and foremost, as a child medical conditions that you can inherit from your parents can be mental issues, diseases that affect the brain, for example Epilepsy, this can be inherited directly from the parents because it affects the blood and it can be passed to you, then you are born with it

**M:** **Any other response? ……… Since we don’t have any response, let us continue and go to the next question. What do you understand by the term DNA? Are we listening? What do think DNA is? Let me repeat the question. Question three says, what do you understand by the term DNA?**

R1: Sometimes as a person who has inherited a particular gene you may not know it immediately but from the people next to you, they can see and say that this child has inherited something from his/her parents because personally the way God has created me people can tell that I have inherited something from my parents. Another way I can tell is that earlier I talked about mental illness. If my parents suffered from mental illness, I will also experience that condition the same way my parents experienced it because all along I have been living with them and I have seen how they experienced that condition and so if one day it happens to me they will say I did not inherit this disease from any different place apart from inheriting it from my parents; that is what I can say.

**M:** **Any other response concerning DNA. This has become difficult we have no responses**

R3: In my own understanding, for you to know that you have inherited some DNA from your parents take for example me, because these things can be inherited and they are in the blood, sometimes my parents my mother may be ill mannered, doesn’t have kind words, so those are things that you can inherit from her and sometimes in the future you could as well do the same things that she did so people will say; “this child has inherited the mother’s behavior”, that is how I understand this

**M:** **Any other response?..... If there aren’t any more responses, let us continue. Question four DNA has smaller components, are you aware of those small components? DNA is a big thing and it has smaller components, do you know about them?**

R5: I don’t have any idea on that.

**M:** **No idea? He has said he doesn’t have any idea on that, what about others?**

R6: No idea

**M: Raise your hands so that I can know who wants to say something.**

R10: I don’t have any idea

R2: I think genes can be among them

**Explanations**

**M****: You think that one the components of DNA are genes. Okay for now our question stops there, let me briefly tell you how it has been written here; “Thank you for your response”. Let us now discuss about these two things here. All living things come out differently and their children inherit from them, for example our skin colour we inherit from our parents, the way your skin is shows that you have inherited from your parents, your height- if your parent is either tall or short if your parent is short you cannot be tall because this is inherited from parents to their children those are the things we get in DNA. are we all listening? So that explains how DNA is. They also defined DNA as a group of cells in the human body that have come together as a man and a woman who then give birth to a child then they pass these traits to their children but they don’t pass it all. For the mother and the father also come and they pass it to the children this means that the child will inherit from both parents. They also defined genes as a part of DNA that makes up what children are supposed to inherit from their parents during reproduction. They also said that genes have cells that define what constitutes of parents it has small cells that are inherited from parents to children, they have explained that genenom are cells that live with other cells genenom are also cells that produce knowledge that are inherited from the parents to the children for example if your father was a very intelligent person the genenom transfers your father’s intelligence and passes it the child. Like in Acholi here they say this person has inherited the father’s dullness or the intelligence of …. this means that it is the genenom passes on traits to you the child or like for us it is passed from us to our children. So if you are a woman and get a man let’s say you are intelligent and you get a man who is not intelligent it means that your intelligence will not all be inherited by the children, the husband’s dullness plus your intelligence will mean that the child will not be as intelligent as the mother and the dullness of the father will not reach that level for the child which means the child will be a mediocre. Are we understanding? That is how it has been explained. They have also mentioned here the things that make up decision making people around you your parents they also added that making up your mind to test your DNA and also finding out the results. You can make up your mind and go to the doctor and test your DNA. When you find you can go to these small hospitals whereby your results let you know how you are. Are you understanding? So that is how they have explained. The topic says; Feedback on the results of the DNA test. We want to know your understanding of DNA in regard to your tribe, your culture, your religion and also according to your own understanding. Are we together? Let us then go to question five. I know that we now have an idea of all this. We want to know your understanding in regard to your tribe which is Acholi, the Acholi culture, then religion, and your own personal understanding on these questions. Question five is all about testing DNA. Question five roman number one says, “do you have any knowledge on testing of DNA?” Anybody here has an understanding on testing DNA? Has anyone heard about testing DNA from somewhere?**

R3: I understand that they test DNA

**M: Any other response?**

R4: Does it require us to respond by saying how the test is carried out, or …?

**M:** **If you probably have a little bit of knowledge on DNA**

R2: So, if we respond that we have knowledge about it, does it require us to give some examples?

**M:** **It doesn’t stop you from giving examples, if any**

R5: I might also have an idea on testing DNA. For example, if anyone passes on, they can test the DNA to find out the cause of death.

**M: Any other response?**

R9: In my own understanding DNA is tested, this is because of one thing, for example when two people or parents are fighting over a child let’s say the child was exchanged with another child in hospital to confirm who is the parent of the child, the doctors make sure that they test the DNA of that child so that they can tell whose blood exactly is in that child’s body. Secondly, there was someone who defiled a child but the person was really denying the allegations, the sperms were removed from the female reproductive organ and it was tested to find out if he really did it. That is my little understanding on DNA testing.

**M: Any other response?**

R10: In my own understanding, sometimes a woman might be doubting herself then she might have go out and pregnant with someone else other than her husband DNA will then reveal the truth in this case, that is how I understand.

R4: In my own understanding too, I know that DNA can be tested and if you want to know your DNA it can be tested. For example, if I have a child and I really don’t understand the way the child behaves, I make up my mind to have the child’s DNA tested for me to understand why the child is behaving like that.

**M:** **Any other response? In addition to this question, do you have a neighbor who has maybe tried to test their DNA? Or how did you feel during that time when you wanted to test your DNA? Earlier on we asked, do you have any knowledge on DNA? You replied saying you have knowledge on DNA. In addition to that question, we asked if there maybe someone close to you who has tried to test their DNA? Or how did you feel when you tested your DNA?**

R8: Nobody close to me like a neighbour has tried testing for their DNA. In my own understanding in any situation whereby they are testing for DNA, I don’t see anything wrong with that but I would like to know who I am and what kind blood I have in me because everyone has their lineage it depends on how the so for my own good I want to know which lineage I come from so that I can understand well because some people do things that don’t go hand in hand with that particular family lineage or clan which leaves people in doubt, I don’t have much to say.

**M: Any other response? Since we have no response lets go to question five roman number two. Sometimes in life people may test for DNA to find out whether they have some bad luck, or a particular disease or other complications what do you think is the essence of testing for DNA because of diseases that they are prone to. Is it clear? If it is clear please answer and if it is not clear, I can repeat.**

R5: Please repeat

**M: I Repeat? They say that sometimes in life people test their DNA to find out whether they have some bad luck of getting diseases or other complications. What do you think is the use of testing for DNA for your family so that they are aware of the disease that they may be prone to?**

R6: I think that testing for DNA is not bad. It is important because in future you should know what you are going to pass through. For example, if you want to know the health status of your child and you think that in future the child might have some health complications it is important for you to know that in future this child might have complications so this helps to see that such issues are worked on so that in future it doesn’t become a problem

**M:** **In case you have something to say please say it according to genetical complications for example complications like Sickle cells. Is there any other response? In case you test your family members especially you and your wife**

R2: I think testing is very important because it lets us know the kind of life each and every one of us at home is living especially health wise and any complications can be detected

**M:** **Any other response?**

R5: In my own understanding I think testing for DNA is very important for me and my husband if we make up our minds to test and find out what our gene is made up of it helps know what kind of children we will end up giving birth to. We will know that the child inherited this maybe say from the mother and father this will help have no doubts on where the child might have gotten the complication so to me this testing is very important.

R1: In my view, it is important to test DNA because if for example a child is diagnosed with sickle cell it will automatically be known that the child inherited the disease from say the father so this will help with the fast treatment.

**Return of results:**

**M: Let’s go to question six which says, what can make you take the decision to participate in testing for your DNA?**

R3: If they come and teach me very well on what they exactly they want to do plus let me know of the cons I can accept to participate in this DNA testing, it will also let me know my health status and they will also help me in case I have any health complications.

R7: I think my participation in the DNA testing is important because I can gain knowledge of which I will go and share with the people left back at home on how they can be helped

R8: I think if they teach me and I understand very well however for me to make the decision to do the DNA test, those people teaching the community should sample the DNA test on themselves first

**M: Let me repeat the question again. It says what can motivate you to make up your mind to take part in this research of DNA testing without fear. they also added that I can the following, Confidentiality, sharing of results with family members, the use of your body parts to do the research, sending out your body parts. So if you have any more responses please tell us**

R10: Using my body parts, I am not interested. If they are to teach me my blood group, I understand but if it is something else am not interested.

R2: I also think that if possible the machines used should be brought here and then you can test but you have to start with yourselves, like a number of you then after a few hours you can start on us. If it is ok, all the community members will come and participate but if the sample is to be taken miles away you know if they take your clothes we believe they you can end up disappearing from your bed.

R6: I believe this could be a good initiative. The only issue that is bad is the issue of using a piece of your clothes or taking part of your body. Take for example we have come here and neither your clothes nor your body parts were cut but then when you go back and you have patches on your clothes and your skin, people will ask you what has happened to you and then you reply that the doctors are going to test my DNA, with the belief we have they will start saying those are your own plans. Ever since we have visited hospital there is no single day our clothes have been used as samples. How did that start? You might end being caned and asked to go back and bring your clothes. So this is going to be hard. It will be simple if people have knowledge, without knowledge this research will not take place.

R3: I feel the knowledge you have brought is good. Sometimes we understand and sometimes even us who are here will not understand. I am kindly asking you to sensitize the community and they will also understand that part of their bodies can be taken for testing if there is a strip for testing for DNA they should bring it to the community so that people can see how they are going to be tested. For the issue of picking the sample and going with it, the percentage of people who will do this test will be small of which I am also not going to test. That’s the truth.

**M: Let’s get this straight. This DNA testing is not going to take place now. We are just doing a research to gather people’s ideas. Just let us know of your ideas. The next question says What if this DNA testing is to be done in the communities to find out diseases affecting people, will you allow to participate in this research?**

R8: If they teach me well and I fully understand how this research works I can accept to participate.

R5: If they also tell me very well whereby I also fully understand this research I can accept to participate so that I can be an evidence to the community to let them know that is not a bad initiative after all so that the research runs smoothly.

R9: Let’s say I accept, now those samples that they pick and go with, how long does it take for the results to come back?

**M: It depends…… it can take about a week to two. Any other response? Let’s go to the next question. Now that you have accepted to be in this research, we want to know if you will be interested in knowing the results of this test. Do you want the results to be brought back to you?**

In chorus, yes, it’s very important

R6: I think these results should be brought back to me so that I can know how my blood is.

R7: The results should be given to me directly. In case they find any medical complications the person who has brought the results should explain to me their findings and also if possible bring medicine and prescribe for me how to take the medication. If you give to the results to someone else the person will begin telling people behind my back how my condition is very worrying and bad.

R10: Earlier you talked about confidentiality which automatically means that in case they pick my sample for DNA my results will definitely be given to me meaning that whatever it is it will be between me, the doctors and the people carrying out the tests.

R8: It is important for me to know these results. If they see that it is complicated they could explain the results in the presence of both my parents and those results if they take a day that’s toolong. The period that they can work on those samples is one hour whereby I can get the results immediately. This issue of taking the sample upto Kampala, I don’t want.

R2: It is good for someone to hear their results personally because the person giving the results can guide me on which hospital I can go to and get medication.

R4: It is not right for a doctor to show my results to someone else to bring to me. This is because that person doesn’t have the experience unlike the doctors who can counsel me. Also if the results are bad then he will prescribe for me how I can swallow my medicine but if they bring someone without experience I will be hard for me to get my results. Also if the results take quite a duration of time then it’s not possible for me to take the DNA test.

**M: In addition to the question that all of you have answered, can you give your results to your family members or to close relatives, let’s say your results have been given to you personally, can you also show your family members or close relatives these results?**

R7: I feel it is right to show my results to family members like my wife so that she can get to know about my condition.

R3: It is right for to show my results because that tool that is used to get the disease we share so if I have the disease she also has it. I feel it is the same.

R8: I also feel it is right to tell my parents because it gives my brothers and also my wife the opportunity to also go and test in case I turn out to be positive of any illness so that other children don’t inherit the diseases too.

R2: It is right for me to show my results to my family members because they can easily offer some words of advice to me.

R1: I feel it is right for me to tell my parents my results personally, not the doctors doing so because the doctors picked my sample alone and not with anyone else. So I want to know my results personally then I go ahead and inform my parents of my condition.

R5: I also feel it is right because this DNA once tested it doesn’t necessarily mean that they are only testing for diseases, they test for many other things. I have people in my circle whom I trust, they not necessarily relatives, I can’t easily tell them of my results and they advise me on what to do.

R6: Personally, my family members should know about these results. We very well know these results turn out to either be good or bad. When the results are bad of course I will not want people to know about them immediately, it will be hard to tell my parents immediately I receive my results and yet sometimes they would want to help me as soon as possible. So am asking these people carrying out these tests to move with a team to pick my parents and I (this should be done if my results are bad) and take me for counselling so that I can live a normal life. Trouble comes in if I am just left like that without any help.

**M:** **Let us continue. Number nine, the headline says, “Giving back results.” So the first question says, if you accept to participate in testing of DNA, what is that that makes you make up your mind to take part in the research?**

R9: What makes me make up my mind to take part in the testing of DNA is teachings like these ones, this will make me make up my mind to take part in the research

**M:** **Let me first repeat the question. If you accept to participate in the testing of your DNA, what is that that motivates you to make that decision. They also added confidentiality, giving your DNA results to your family members, using your body parts for research, sending your body parts for other researches. We want you to answer along those lines. I repeat, if you accept to participate what motivates you to make that decision.**

R5: I can accept to participate in the testing of my DNA. I will do it because it will help me to know of any underlying medical conditions because there might be some conditions that am not aware of, so if I accept I will get to know whatever is going on in my body and also I will get a way of helping myself.

R3: I will accept because they would have taught me and I would have understood very well what they want to do. This will allow me to make up my mind and also I will know exactly what to do and also know what exactly is needed for my life.

**M:** **Any other responses?**

R10: What will make me make up my mind is in case they carry out the tests in my presence and also my results given to me immediately then I will make up mind to participate but if they take the tests far, then making up my mind is tricky.

R6: I accept to participate but let me take you back and say that making up one’s mind is not something easy. I can participate because I heard that they enforce confidentiality, and also they can take your results to your family members, this can encourage you to make up and your mind and participate because if the results are out, the doctors can keep my secret. In addition to that, it might also be hard in a way, let’s say results are out and you are told you have mental issues, it will be a big blow to you and you will always keep thinking about it. even if the disease has not escalated, it will automatically start because you are always thinking about it, so sometimes it is hard to make these decisions.

**M:** **Let’s go to the next question. Should your DNA results be given? If so, to whom should it be given? Should it be given to you the patient, or to your family members, or to community members?**

**R1:** Yes, it should be given. It should be given to me the patient so that I can know exactly what they have found out.

**M:** **You have not answered one question which says, should it be given to you personally, in written form or a phone message? Which method should the results come in?**

R6: They should not send a message. Come to me direct with a small slip and come and explain to me. If it is a message it is tricky you know messages have a lot of fraud.

R3: The results should be given to me immediately without even taking a lot of time because it leaves me with the information because if they carry out these tests and they delay that side, you can be on tension and unhappy because your heart is not settled, so these results should come back to me personally. The doctors should be the ones to bring the results themselves because someone can send a wrong message.

R5: The results should be given to me personally because it is me supposed to tell my parents and also I would want it in written form because the records can help me in future, let’s say my condition becomes worse, I need to show those results at the hospital. If you get your results via message you can’t go and show a message to a doctor so I feel it’s better to receive in written form.

R8:  To me I think it depends on the type of disease because sometimes it might be a non-life threatening condition or it can be like epilepsy which doesn’t go hand in hand with noise so the people back home know how to handle me and if they decide to give me the results alone at least my parents have to be there and also they should put in the records so that incase another disease comes in the doctors will have idea on how to help me.

**M: Any other response?**

R9: I was away so I need to also understand the question.

**M:** **Ok, the question says, should your DNA results be given? If so, to whom should it be given? Should it be given to you the patient, or to your family members, or to community members? If given to these people, what method should be used?**

R6: At times I might not have the opportunity to be around people close to me during that time of testing. My results should come to me personally. The method they should use, I know the way doctors work they start with counselling, they can counsel me and listen to my ideas when am still upright. They should give me direct and not phone message. Since they have picked my sample let them bring my results to me.

R7: After testing, personally I should know my results. There are some diseases that affect the brain, if I find out that I have mental condition, I need to go to my parents and such diseases in case some people are not aware, you can end up harming people so if my parents are aware they can inform the local leaders of my condition so that people are aware because when it comes to mental issues, I am not ashamed if people find out because they will also know how to help me.

**M:** **The next question says why should your results be given to you?**

R10:  The results of the DNA should be given to me because it was part of my body that was removed, it was nobody’s body part, it was mine.

R2: I think the results should be given to you because by the time you went for the test you wanted to know your health status so the results should be given to you so that you can know about your health status better.

R4:  When I go to test I go because I know that I want to know my health status, so if am tested and they don’t give me my results it almost like I have not done any tests, so if I get tested my results should be brought back so that incase I have any underlying conditions I can look for help.

R9: For any test carried out, the results will either turn out to positive or negative, and for any underlying condition the results will turn out positive, so these results should come back so that I start living a new life.

**M:** **the next question states, which method should be used do that the results can reach you?**

**R8:** If my samples were picked from home, that means that my result should be brought home and also before giving me results they should counsel me but they should not just give me the results abruptly. My mind should be settled before giving me my results.

R6: The team that picked the sample, for instance we learnt of your plans to come here three days ago that you were coming here, so we can be given prior notice or a form stating the date the results will be brought back or we can use phones, when filling in those forms we write our phone numbers that on such a date please go to your health Centre you will get the team that did the tests, you will find your results there. Or they can come home pick samples one by one, it will be right to counsel me together with my parents so that they can know what to do, those are the ways our results can come back to us.

**M:** **When the results are given to you, can you give these results to community members or close relatives?**

R8: It depends on the type of disease, if it is a disease that I can survive with by taking care of myself I feel it is ok to keep it to myself but if it is a condition that needs people’s like me getting lost, then the community should know about it.

**M:** **In regards to testing for DNA sometimes the results might turn out to be different from our expectation, do you think if the results turn out to be different is it right to give you those results?**

R2: The results should not be given to anyone else apart from me.

R1: If the results come back showing that I have an underlying health condition it is important to let my parents know

**M:** **If you feel it is right for the results to be given to you, what method do you think the results should be given to you.**

R4: I earlier said that if the results come out bad my parents and I are taken for counselling and the results are given to them in my presence.

R7:  I think it is right to give results to that person whose sample was taken in front of their parents especially those who are still young and if you have a partner it is important for you both to know these results so that the other person can be there for the person whose results turned out positive for a medical condition. I feel it is important for the people close to the person whose tests have been done to know about the results.

**M:** **Why do you think your results should be given to you in front of your parents?**

R6: If the results turn out to be positive of a medical condition this will give me the opportunity to start looking for a solution.

**M: Any other response?**

R2: So that my guardians can offer help to me where necessary.

**M:** **Any other response? Let’s continue. Sometimes results of DNA might s reveal new information to some families or even the community, what do you think can be the outcomes of such results? For example, in a home where the parents carry the sickle cell genes, they get tested and all that, what would be the reaction of the family members since they can easily pass on the sickle cell gene to their children? Or what would be the outcome of these results?**

R3: Start looking for solutions and also seeking advice from the experts of sickle cell so that we can see what to do. It is something bad because it is to help the future children so that they don’t get infected by sickle cell. Since sickle cell is an inherited disease from parents, it is now the time to start looking for possible solutions so that they don’t pass the disease to their children.

**M: Any other response?**

R1: Before the results are given to this person, the first thing they should do is counselling that person so that when the results come they are not so heartbroken. Also in evert result, there is a positive or a negative. We all know that sickle cell is inherited from parents to children so incase my results turn to be positive for sickle cell gene they should help with medical advice so that my children do not get infected by this disease.

**M:** **Do we have any more responses? In regards to the results of the DNA tests, what do you think should be done to avoid violence in a family or home?**

R10:  it would be good to pick samples from both parties so that when results are out both of them are aware of their status. If they find that one person’s result is good and the other is bad, this is something just inherited from our ancestors so it should not trigger any violence because it is not something someone would wish for and the time you get it you are not even aware so they need some counselling to let them know that it is something inherited.

**M:** **Any other response? Am waiting. We need to wind up.**

R9: Violence in homes is common and it is not only caused by one thing but by many things. There is only one solution to this issue like she said, it is sensitization. The two parties should be sensitized and counselled. Their samples be picked together and their results should be brought at once, together. After they should discuss the way forward together. It is not right for only one person to test then come and inform the other of the results, this will bring violence. This is for couples living together. For non-couples, if they find a child who is still under the custody of their parents, they should counsel them as well and look there will be no violence but if results come back, let’s say the man went for testing alone, and then informs the other, this will lead to violence.

**M:** **Do we have any other response? The last question says, in regards to everything that we have discussed, do you have any suggestions, this is the time to air out your views in relation to DNA.**

R5: Since you have already started it should not stop today you should come back here time and again so that we the community members so that we start we can know how our lives are. But if you come today and then disappear it doesn’t look good. I ask you to come back in future.

R8: This program should not stop here, when you go back to Kampala come back to sensitize the bigger number of the community members while using us here to convince people back home that this imitativeis good because according to me DNA is very good since some children here are not staying well with their parents because they believe that these children are not theirs. Violence is there in homes where you find that a child has been denied by the father, maybe my wife got pregnant with someone else, this is not my blood, this brings violence in homes, so please come and sensitize people so you start giving DNA to reveal that this person truly comes from this family, from this clan so that people are aware of the situation to avoid unnecessary violence amongst themselves, that is my suggestion.

R4: If this is a government program you should go back and tell them that we welcome this program the way it is, it has so far been good and we have listened carefully. Most times when we go to hospitals we always hear things like DNC, DNC, and yet we don’t have any idea on what it is but right now we have an idea, so am asking you to take your time and come back so that this knowledge spreads to other members and also my second concern is can this program be voluntary work where a few people can be picked and they teach other people especially when you are not there so that when you come back other community members already have an idea and for you just refresh their minds, or will it be just like that?

**M:** **If there are no more suggestions or ideas we shall stop there. I want to thank you for your ideas. Our session ends here, thank you for sparing your time to come and share ideas so that these ideas can help Ugandans and people of Acholi in regards to our DNA. We don’t have much to say, our meeting ends here, thank you for your time.**

**M2:** **Thank you very much, wapwoyo matek. Thank you for your time, we value and appreciate your contribution and we will do our best to make sure that this contribution is really represented in our report. Thank you very much.**

**FOCUS GROUP DISCUSSION 008 GRASSROOTS COMMUNITIES ABOVE 35 YEARS, NORTHERN**

**M: We are going to discuss, we shall ask you questions and you respond, we ask questions and you give us the answers. Are we all listening? So the first question says, what can we inherit from our parents? Are we all listening? What can we inherit from our parents, for example myself, what can I inherit from my parents? That is my mother and my father. What can I inherit from them from birth or in the process of giving birth to me?**

R11: First and fore most we can inherit our parents is their behavior. Secondly if your parent is short or tall, sometimes you can inherit that and you can either be short or tall. And also you can inherit how your parent behaves. Thank You!

M: **Any other response?**

R7: The second response regarding what we can inherit from our parents is let’s say your parent has paid you in school, this means that you are inheriting this from your parents

R8: We can also inherit diseases like HIV from our parents. It can be transmitted the parents to the child.

R5: Another thing is if your parent is a witch, then a child is capable of being a witch.

M: **You mean the parent’s behavior**?

R6: Yes, their behavior.

R4: A child can inherit their skin color from their parents. It can either be light or dark skin complexion.

**M: Any other response?**

R1: Another thing we can inherit from our parents; let’s say your parents have long legs, a child is also bound to have long legs, if the parents have big eyes, the child will also have big eyes.

R10: Let’s say for example your parent is a thief, as a child you can also inherit that trait and you also become a thief. Thank you!

R12: Sometimes if your mother or father is a witch, a child can inherit that from his/her parents.

R8: Another thing is if yourfather is a violent person who beats his wife, a child will see all that and also start doing the same.

R9: One of the things one can inherit is the blood group. If your parent for instanceis of blood group O then you can also inherit that blood group O.

R7: My second answer is that if your parent has not been teaching you well, then you as a child won’t be clever, you will be as dull as your father.

R6: Another thing we can inherit from our parents is, if your parent is hardworking, then the child can also be hardworking. Thank you!

R11: There are very many things we can inherit from our parents. One, if your parent is a thief, he will teach you with his habit of how to become a thiefand the child will also become a thief. Secondly if the parent is a witch, then the child will also become a witch.

R2: As a man what I can inherit from my parents, I am a man standing on my feet with religiously the way we were taught, like as a person I should respect people, I should do a lot of things especially religion and respect.

**M: Since we don’t have any other responses, we continue with other questions; the second says; what health issues can we inherit from our parents? Question number two says; what health issues can we inherit from our parents?**

R4: Some of the health complications we can inherit, you know sometimes at child can be born with a HIV that is transmitted from the mother, if the mother has HIV the child can easily get HIV from the mother.

R9: Another complication we can get from our parents is the time when the mother was pregnant and she wasn’t eating well the child will be born malnourished, or even when the child is born and is not fed well, then the child will be malnourished.

R10: One of the health complications we can inherit from our parents is if my parent is short, I can also be short, a condition known as dwarfism. This means that you are short and it is inherited through the genes.

R3: Another health complication we can get from our parents is;let’s say the parent has syphilis, the baby can easily get syphilis from the parent especially if it is not treated well.

R1: If both parents are drunkards, it means that the children will go hungry because the parents will not have time to cook and also work in the garden.

R12: One of the health complications we can get from our parents is sickle cell disease, one can be born with sickle cell. Another one is we can see an example from Ojara’s (name of someone) children they call them Albinos, if one’s blood is not strong enough, you can give birth to such children.

**M: Do we have any other response? If there is none we continue to the next question which says what do you understand by the term DNA? These small cells called DNA, what do you know about them? What is DNA?**

R8: DNA is “Deoxyribonucleic acid” that is in the cells that are joined together and that is where characteristics or how people behave are found in the genes.

R5: For me to know or understand what it is, I should make it a habit to go to hospital so that I can know if I have any health complications or what kind of genes I have.

R7: I understand DNA as small cells that if I give birth to a child, I pass it on to the child and if I want to know if it that is my child, they pick my bloodsample and will be tested to confirm if that is my child. That is how I understand.

**M: Any other response? Mothers? Let’s continue. Question number four says, do you know any other small cells apart from DNA? Do you have any idea?**

R9: The one that transports energy, that one that gives energy. There are others that are in cells, they are very many they give energy, they let you pass stool, all the things people do are in cells

R11: The cells that I know that are different from DNA is RNA which is also known as Ribonucleic Acid. Thank you.

**Explanation**

**M: Any other response? They have written something here to make people understand. It is written, thank you for your ideas. Let us discuss two issues. All living things in this world have different characteristics or traits which they pass on to their children. For example, your skin complexion, eyes, your height, these examples are found in DNA parts that control our behaviors. They also went ahead and defined DNA that DNA is a group of lives of people who have come together that take control of anything that comes to life. They also went ahead and defined genes as a group of cells that control what parents should pass on to their children during child reproduction. Genes have cells that store small traits that come from parents. They also defined Genome as cells that are like the way someone behaves. It is something that collects knowledge for life. They also went ahead said things that determine decision making. The knowledge on DNA, your family members, the people who have given birth to you that is man and woman, your decision to take part in DNA testing. Doctors have the knowledge on preventing any harm from happening during this DNA testing process. If you want to take part in this test the doctor has knowledge on preventing any harm. Anybody taking part in this testhas the right to give the results to family members. Are we all listening? Sometimes giving results that are good help in decision making. For example,sometimes results don’t let peoplemake up their minds. We want to know your viewsin regard to your culture, your clan or even your religion. You can also tell us any other view that you have.**

**You can tell us either how your culture or your religion or even your own view. So question number five says, do you have any views in regards to DNA testing? That is the question, so we want you to tell us if you have any idea or if you don’t have tell us you don’t have any idea. Also if you have any idea, have you heard of anyone who has had their DNA tested? So it all goes back to you. Do you have any idea in DNA testing?**

R4: I don’t have any idea on how DNA is tested.

**M:** **Or have you heard that DNA can be tested?**

R6: Yes I have heard.

R9: I have an idea that it is mostly done in Lacor hospital but another hospital where it is mainly done is in South Africa where they test for DNA, but because of poverty we can’t test our DNA.

**M: Any other response?**

R11: We don’t have any idea.

R10: We don’t have any idea.

**M: Any other response?**

R8: We have an issue of lack of money to go and test in far places.

**M: Do we have any other response? If we don’t have any response, let’s continue. Question five roman number two.**

R7: I want to answer.

**M:** **Okay, you can answer**.

R3: We do not have any idea on that because the hospital is far from here and alsowe don’t have the money that is supposed to help us to go and test DNA

**M: Question number two says, sometimes in life people can test for DNA to see if they have any bad luck of acquiring diseases or any other complications. What do you think about testing for DNA so that your family members can get an idea on the diseases that can affect them? Let me rephrase.**

R6: To me, I should test all my family members so that they can know their DNA to know their chances of getting diseases related to DNA. For example, sickle cells, albinism. I have to know in case we have these in our family so that we can know what to do early enough but these are things we can’t do here.

R1: I think it is good for me to test my DNA because it will help me plan and know the health status of my family.

**M: Any other response?**

R5: You know testing of people should be done time and again but because of poverty as earlier I had said, it is hard. They should bring good machines and then they test us on a monthly basis so that we can have an idea on what is happening in our bodies.

**M: Do we have any other response?**

R8: I would really want for my family members to test for DNA but because of no money and the distance to the hospital prevents me from doing so. I really want to test. Thank you.

**Return of results:**

**M: The next question, what is that that motivates you to participate in this research without fear? this research for testing for DNA. What motivates you to make up your mind without any fear?**

R9: What motivates me to participate in this program without any fear, is the fact that I get to know about my health status. Thank you.

R12: What motivates me is what you people are doing here and also in future when it is now time to draw blood and do the necessary tests is that these teachings will help me understand how my life is.

R10: What really makes me not to fear in this teaching is it is important to know my health status and also if now know that this type of disease is in my body, I should now follow the doctor’s instructions for example like in this health center IV incase tests are done, and it is discovered that I have hepatitis, what should I do, they then tell me what to do so this gives me the energy. This will make me strong.

R7: For us we only listen to doctors, we do not want any witch doctors with their stupid ideas and confusing people.

R1: What makes me accept without fear is the fact that I know what DNA is, or I know what genes are, so I decide to make up my mind so fast so that I can be in this research.

R8: Mine is different in a way that if you people go and you don’t comeback, this leaves us in fear because Uganda now days is different, there are very many weird things in it.

R5: Mine is also some piece of advice to you. One you should teach people because if people are taught on what you are doing, people won’thave any fear on what you are going to do since they know that the main intention of the testing is for health.

**M: The next question says, do you want to know the results of testing for your DNA in case you participate in this research? Do you want to know your results?**

R1: Yes, I want to know the results of this DNA test because it helps me to know the status of my blood and also know my clan too.

R4: It is right because it helps me to know my status which gives me the strength to take care of my children.

R5: On a higher note, I want to know the results of my DNA test and also it would have been very important as we talk now foe the machine for testing to be here because I really want this.

R9: What I want to say, is that, doctors after testing, you write it direct, your handwriting is different, it is not readable so for we don’t have an idea of what we are diagnosed with.

**M: In addition to your answers above, there is another question which says, after giving you your results for the DNA test, can you give those results to your family members, or to people close to you?**

R10: Yes, my results are important to my family members to know but not outside of family because sometimes that is a secret we have in our house.

R12: Yes, it is right because if I have my wife, I should not keep it away from here especially if they find out that am sick, I have to let her know.

R3: My answer is no different from the rest because if I take the DNA test, and I get to know of my health status, it is important to tell my wife about my health status, also, she needs to go and test so that I can also know her health status so that we can live in harmony in my family. Thank you.

R4: It is also important to me to tell my husband or to my family so that they are aware of my health status but they should not tell anyone outside family because it is a secret issue.

**M: Let us go to number 6. The heading says Giving back the results of the tests. The question says, if you accept to test your DNA, what is that, that makes you make up your mind? Number 6, roman number one says if you accept to test your DNA, what is that, that makes you make up your mind?**

R7: What makes me accept is the fact that I would have allowed to participate in this DNA testing and also to know my health status.

R11: What makes me make up my mind is the fact that I would want to know my health status and also in case of any health complications the doctors should save me quickly.

R3: Mine is a quick one, we should just have belief. I should believe what they are doing well and I must accept with love.

R9: What will make me accept to do this is on me, is to know if I have any illness so that the doctors can give me medication so that I can live.

**M: Question 6.2, in case they were doing a research in the community to show that you, your family members and other community members will be with a certain disease, can you accept to participate in this type of research? Let me repeat, in case there is a research going on in your community, to show that you, your family members or members of the community to see that there is a certain disease, can you accept to be in this research?**

R10: I can participate because it is important to do such things so that it can give the government time to see how to help us.

R4: I can accept so that it will give the government time to budget on what medicine to buy to cure this disease.

R6: I will accept. For example, right now we are in difficult times. Times of corona. In case you have from far and there is a rumor that you have corona, we shall truly accept for the doctors to investigate your corona so that you know that we shall not be near you during that time. Thank you.

R1: I accept because let’s say I have a condition lie Albinism and also my wife has that condition, we should not pass those genes in the environment.

**M: Since we don’t have any other responses, if you accept, do you want to know the results of this tests if it ever happens?**

In chorus; Yes, we want to know.

R9: I want to know the results of those tests because I want to know my health status so that if am sick I go to the hospital, if am not I start planning my life afresh.

R12: I want to know my results because if you test for anything you have to know your results so that I can know if am healthy or sick.

R8: I want to know because if I don’t know then it’s not good. I want to know what is happening in my body.

R9: I want to know the results of those tests because if you don’t give me my results I will not be settled but if I know my results, I will be settled.

**M: Now if you have those results, can you give them to your family members, your close relatives or your community members?**

R7: When those results are out, they are strictly for me, my husband and my children to know about these results.

R5: These results will remain in my house; I will not share them out anyhow.

R6: I will also accept for my results to remain within my family members only if she refuses to go and test, those are hers.

R4: I will accept that because sometimes I might have an older child so that he is aware of my sickness in case I have any. He can also take me to hospital. He should know that if am not feeling well it must be this disease, let me go and look for medicine, so my child should know.

R2: For any result for any tests, I will try my best to give it to my wife and children.

R1: I will accept that my wife should know, the community should also know, there are other diseases that can be spread, those near me can even help me if I am weak, my neighbors should also know.

**M: What method do you think should be used to give you your results? Should they bring a letter home, should they send a message on the phone? What method should they use?**

R10: The doctor should first counsel me because sometimes if they found a disease and they just gave me a paper, it can make me unsettled but if the doctor talks me, tells me that we found a disease but take care of yourself, take your medication, this will make me not have any fears. Thank you.

R11: I want those results in a paper because if I fall sick, I can go with that paper to the hospital. They should not send it via message.

R8: I want the doctor to first talk to me and teach me before giving me the results. He can tell me that he didn’t find any disease so you have to take care like this like this. They should not send it in a paper, they should not send a message on phone. The doctors should teach you first. This will make you strong. If they send a message on phone that you are sick, you can easily get shock.

R6: These results should be given in a paper. Sending of message doesn’t work since the phone does a lot. Some people don’t know how to delete messages from the phone, anyone can read messages on your phone. If you are given a phone, you can just fold it and put it there but also keeping it in mind that you should tell your children the content of the results. You can even send a child to go and buy you medicine but it is hard for a child to go with a phone to buy medicine from a doctor.

M7: The next question….

R9: It should be a document that is sealed, what the doctor has written he should explain to me.

**M: The next question. In relation to the results of testing for DNA, sometimes the results can be different from your expectation, do you think these results should be given to you? If the results are to be given to you, how should it be given? Why do you think it should be given like that?**

R5: When my results are out, I want the doctor to teach me, then after they can avail to me my results. If they give me without counselling I can easily get the shock of my life.

R3: These results should be given to me in a paper because doctors know how to read their handwriting and it will also help the way it is. It is also important to counsel me in case the results don’t turn out good. Thank you.

R11: I should get these results in a paper. Before giving me the results, I should first be counselled. Everyone should pick their results on their own, not everyone together.

R1: That now depends on what the doctor wants. If he decides to call me to the hospital, then I will go to the hospital. But he should give me guidance and counselling. He can even come up to home, if I see a doctor visiting me, I can feel very happy.

**M: Sometimes testing for DNA can reveal information that are in families or communities, what do you think can be the repercussions of giving these results? Let me repeat. Sometimes testing for DNA can reveal information that are in families or the communities, what do you think can be the repercussions of giving these results?**

R5: This makes me stronger, my life can go ahead because I would have been advised.

R9: These results if known in the community can let us know that we are from different parts so this brings happiness knowing that these people have all tested, I should also go and test.

R7: There are two things that come out as a result of testing. Sometimes it can bring anger to the person who has tested, or sometimes it can bring happiness, the beauty comes in when one has tested and the results turnout good, this gives the person the opportunity to take care of themselves.

R10: My response is not so different because if I get to know my health status, I can be relaxed and also this lets the community members to come and get some advice from me on how I went and tested, on how the doctor counselled me. The leaves me with knowledge including the members of that area.

R12: That is good because if I get to know my health status and if I am sick, and I have my other friends who are sick we can easily exchange ideas and advice.

R2: It is good to give those results to people surrounding us because if they find that I have a disease that can be inherited, if there is lady interested in one of our clan members, we can easily advice that person that this family has a genetic disease, let’s say maybe sickle cell, we tell her that this disease can be passed on to your children, so this that person the chance not to continue with that relationship.

R1: For me, if I test and find out that I don’t have any disease or even if I have, I can go to the community and advice a friend who maybe is sick to go and see a doctor so that they can know their health status and if they feel like then they will go.

**M: After testing for DNA, do you think the results can end up bringing violence in the home or within the community?**

R2: If I find out that am not sick and my husband is sick, it will bring some violence, where does that disease come from? It means that he is not doing something right.

**M: Another response.**

R9: With results there are two things involved. If the results turn out to be good, this brings happiness, but if the results are not good, this will automatically bring violence in the house.

**M: Do we have any response different from this? If it brings violence what should be done so that this violence doesn’t happen?**

R5: If the results show that either me or my wife is sick, it is very important for the doctors to counsel us because personally my wife will blame me for bringing the disease in the home so it is very important for the doctors to call us to the hospital to counsel us.

R4: Love is a very important thing in the house. If you sit and discus and your ideas are the same then you can now go to the doctor. Your ideas should be the same.

R11: I also want to say that the doctor should talk to both parties that are going to be given results. The doctor should tell them that if the woman has the disease, do this, if it is the man, do this. The two people going to pick results should be strong knowing very well that anytime anyone can get infected with a disease.

**FOCUS GROUP DISCUSSION OO9 GRASSROOTS COMMUNITIES ABOVE 35 YEARS, WEST NILE**

**Awareness/understanding of genes and genome**

**Mod Qn.1: What can we possibly inherit from our parents? *(Physical, behavioral, health conditions)***

R2 female: I inherited my father’s blood type.

R3 female: For me I got my father’s behavior

R4 female: For me people say I look like my father because I got his height.

R1 male: I also got my father’s height and his good heart of always helping others.

R6 male: For me I got my father’s behavior especially being a worker. I am a hard worker and my father was known for being a hard worker, so I believe I got that from him.

**Mod Qn.2: Which health conditions can one inherit from their biological parent?**

R3 female: Like for me I inherited my mother’s good health hygiene habits which are now a natural thing for me.

**Mod probe: Can you give us some of those good health practices please**

R2 female: My mother always kept her home clean, the utensils were always scrubbed sparkly clean and ensured children are always bathed and dressed in clean clothes.

R2 female: I also inherited my mother’s good health practices like keeping the home clean, personal hygiene and always washing foods before cooking them

R6 male: I also think the health education that our parents give us from home while we grow up like; brush teeth, bathe, wash your clothes this way, are some of the health related things that we can get from our parents and if your parents are dirty people, the child will grow up a dirty person.

**Mod probe: What about physical or biological health issues that your parents had that you may have inherited?**

R4 female: I think I got some abdominal pains from my mother; allergy to some foods and the doctors told her it was cancer, which seems to be the same thing I’m suffering from right now and that was what killed my mother.

R5 male: I think I got eye problems from my father because he used to suffer from eye issues a lot like me right now.

**Mod probe: Any other person with another view?**

All: Silence

**Mod Qn.3: What do you understand by the word gene?**

R1 male: I think it has to do with resembling your parents in certain ways for example; I got my mother’s light skin color, my father’s height, he was a tall man and very calm in nature.

R6 male: For me I look exactly like my father who was light skinned and tall.

R4 female: I think mine is also height from my father

R3 female: For me I inherited a wide tooth gap from my father who had the exact gap.

R 2female: I think for me I got my father’s kindness and loving nature from him because he used to love people, so we always had very many people in our home when he was alive.

**Mod probe: Any other person with a different answer?**

All: Silence

**Mod: What do you know about DNA?**

R5 male: What I know about DNA is that it is a medical process of proving the paternity of a child, for example the Doctor will extract blood from both the child and the father for testing and if they are the same, the child is indeed his.

**Mod: Any other person with another view?**

R 6male: For me I think it is just a term referring to the resemblance to a parent for example; you resemble your father in eyes, nose, legs, height, skin color etc, this is my understanding of DNA.

**Mod probe: Any other understanding of DNA?**

R1 male: I have heard when two people are fighting over the same child, the doctor can compare their DNA samples with that of the child and the one similar to that of the child is the actual father.

**Mod Qn.4: What do you know about genome?**

R2 female: How do you define it?

R5 male: In my own thinking, genome is the determination of whether the child is a boy or a girl that is how I understand it, that mathematics that the doctors use to determine the child’s sex, race and even whether they will suffer from albinism.

R1 male: Women should be able to understand these processes since they are the child bearers because they say a mother can tell whether a child is male or female by some sign.

R4 female: Sometimes those signs are not accurate for example they say a male child is positioned in the left side of the womb, but that is not the case always, the accurate way is through medical tests.

R4 female: Among my five children the only difference I felt was that the girl was more active in the womb compared to the boys.

R3 female: For me my boys were always placed in the left side of the womb while the girl was on the right, I have three children now, two boys and one girl.

**Explanations**

***Mod: Thank you for your contributions. Let’s talk more about these two issues. Each species or group of organisms has a unique set of inherited characteristics that make them different from each other e.g. color of skin, eyes, height. These characteristics are usually encoded /incorporated in the DNA molecules present in their cells.***

***DNA is the genetic building blocks that govern all the characteristics of a living thing (e.g. animals, plants, and bacteria).***

***Genes are the elements of heredity that govern what is transmitted from parents to offspring in reproduction. Genes are composed of specific DNA portions or segments that are capable of controlling specific heredity characteristics like eye color, skin color height etc.***

***Genome is an organism’s complete set of DNA, including all of its genes. Each genome contains all of the information needed to build and maintain that organism.***

***An organism has one genome, but it has thousands of millions of genes in that particular organism.***

***Ethical issues***

***Your genetic information can be similar to that of your immediate family, and blood relatives. Your decisions concerning testing and what to do with the results may affect them as well.***

***Health professionals are ethically responsible to prevent harm to those involved. The individual tested also has responsibilities and obligations and should appreciate the shared nature of genetic information within families.***

***In some cases, genetic tests provide reliable and accurate information based on which decisions could be made e.g. … while in some cases, it is not possible to get definitive results. Prediction of a potential condition or disease may not include the severity of the condition or the age for the onset of symptoms. An individual is much more than the sum of their genes: the individual’s environment can modify the expression of genetic messages to the body. Many health factors are not genetic.***

# Attitudes towards applications of modern genetics and genomics

We are going to discuss specific issues to do with genetics and genomics. We would like to hear your views from the stand point of your ethnic group or culture and from your religious back ground. You could also tell us what you personally think.

## Genetic testing

*A genetic test examines your DNA, and can reveal changes or variations in your genes that may be associated with an illness or a disorder. A genetic test can be arranged by your doctor or health clinic.*

**Mod Qn.5.1: Who among you has gone through a genetic test? What was your experience with the process of getting the test?**

All: Silence

**Mod probe: It could have been one of you or any one you know.**

R1 male: I did the test in the hospital

**Mod probe: Did you receive the test?**

R1 male: Yes I did, because I had pain in my chest

**Mod probe: Let me ask this; have any of you under gone blood test with a parent to prove paternity?**

Chorus: No, with mumbled discussions

**Mod: I think we are confusing medical tests with genetic tests.**

R5 male: Is there a difference between the tests for operation and genetic test?

Chorus (mod, other respondents): Yes

**Mod probe: So there is none of you who have under gone a genetic test?**

Chorus: Yes

**Mod Qn.5.2: What is your view on bringing genetic tests within your reach in the communities so that people with a family history of serious genetic diseases can find out if they are at risk?**

R6 male: It is a good thing because there have been cases of domestic violence because of a man doubting the paternity of some of his children, such would help solve some of these problems causing violence in the homes.

R2 female: It would be good because some times when these young girls go and mess up and get pregnant, if the girl identifies a man as responsible, most of the men deny running away from responsibility, it would help to know the culprits.

R4 female: I fact this has happened to me before; my husband denied my second child saying I cheated and when we went to the hospital to prove, their DNA was the same and he even did not apologize for accusing me of adultery.

R3 female: For me I think this would be very helpful because in this community especially there are many people who have grown up in their maternal grand parents’ home because they were denied by their fathers so the mother’s people suffer to look after them and we still don’t know their fathers.

R2 female: For me I would like the tests to be brought here because I have some health issues that I have not seen on my parents, my mother is disabled but I am not, so I want to prove whether I have their genes or not, whether I am their child or not through those tests.

**Mod probe: Does every one think it’s good for the service to be brought within and is it for the same reasons you have given?**

Chorus: Yes

**Return of results**

**Mod Qn.6: If there was a study on GG that could potentially reveal that you/ your family or community is susceptible to certain diseases which are very difficult to treat, would you consider participating? Would you want the research results if you participated? Would you be willing to share the results with immediate family, blood relative, and community and beyond?**

R6 male: Yes I would accept.

R2 female: Yes I would

R1 male: Yes I would be willing

R5 male: I would accept

**Mod probe: Would you want to know the results of your test?**

Chorus: Yes

R6 male: Obviously I would want to know

**Mod probe: Is that what we all agree?**

Chorus: Yes

**Mod probe: Would you tell your family, other community members?**

Chorus: Yes

R5 male: Yes it’s necessary to tell them

R3 female: They will have to know

**Mod Qn.: What can convince you to accept to take a genetic test with confidence?**

R1 male: For me it would be the results of my test which would be giving me true results that I can use to help my condition and that of my family.

**Mod Qn.: If you were told that your family line has a hereditary disease which needs to be tested for treatment, would you still be willing to take the test?**

R2 female: I would accept because if a disease has been realized in our family line and there is hope for a solution after testing it, it will help us otherwise we shall never know the disease let alone the cure.

R6 male: Since no one chooses what family line to come from, I would accept if I’m already connected to the family.

R4 female: These things are there, like in some families’ right from the great grandparents, at least one person dies of a strange disease but the doctors just name minor illnesses as the cause of death yet there is a major hereditary illness. Like in my family we have a history of abdominal pain from which my grandmother died, my mother but all the doctors said was liver failure.

All: Yes they are there.

R1 male: Honestly, I have never heard of a family lineage dying of liver failure may be things like cancer

R4 female: For me I believe some of those illnesses are as a result of malice on a whole clan either through a curse or witchcraft.

**Mod Qn. Would you want to know the findings of the test? Why?**

R5 male: Yes because I would want to find out the truth instead of guessing.

R6 male: My reason would be to know the facts of my health condition also or that of my partner.

R6 male: I think the main reason is to know what diseases we have in our families, how they are acquired and how they can be cured.

**Mod Qn.: How would you want to find out the result?**

R3 female: If it were possible, I would want to see the nature of the disease through an image or explained to me thoroughly.

**Mod: Who should be given the results?**

R4 female: The test should be done and the results given to the care taker not the patient, not the family members and not the community and the condition should be properly explained to the care taker.

**Mod probe: And how should the message be conveyed to you or the care taker, if you are found to be sick?**

R2 female: Of cause as doctors they know how to describe the results of a test; they should be professional in breaking the news to me.

**Mod probe: Any other view?**

R3 male: There is no other view, the doctors know the best way.

Chorus: Eeh (yes).

**Mod Qn. Why do you think it is necessary to give you the result?**

R1 male: To help me know what the illness is and whether the complication is from my mother or my father, so that I can alert them and see how to protect my children.

R2 female: So that people can clearly know the actual cause of the death of a person, not that they are left to imagine.

R6 male: We know that these diseases could have come from the ancestral line of our parents, so knowing the result is good because you can be able to tress whether it is coming from your mother’s line or father’s line and inform them to protect the next generation of the family.

**Mod Qn.: How should the result be given?**

R4 female: The doctor should call the care taker on the side in a private space to give him/her the result,

Chorus: Ahaa, laughter.

**Mod: Do you accept your result to be given to your family members?**

R3 female: Yes, they have to know because some of them may have the same issue.

Chorus: True

R5 male: That is a family health issue which can affect any member, so they have to be told the results.

**Mod Qn.: Should any extra findings be told to you, your family and why?**

R2 female: Obviously

Chorus: Yes everything has to be revealed there and then

**Mod probe: Why do you say so?**

R3 female: For the same reason that you know what is causing you pain and find a position if possible.

R4 female: Even better so that there are no more doubts because if others are not detected, you may still have the pain and start wondering what is happening to your body.

Chorus: Sincerely, laughter

**Mod Qn.: Why is it important to give your results?**

R1 male: It means heath complications should always be openly shared in the family so that everyone is aware and informed.

R3 female: We will normally share with others to find support for the solution needed.

**Mod Qn.: What is the best way of giving the result?**

R4 female: The doctor speaking quietly to the care taker not publicly

R6 male: In a very private room with no listeners

R2 female: If they say someone has a disease, everyone will want to know and stat spreading the news like wildfire and yet even the one who is suffering from the disease has not yet been told.

All: Laughter

**Mod Qn.: Is there any other issue concerning what we have discussed that you would want to share?**

R1 male: For me I have nothing else to talk about

R4 female: Mine is a request that these tests should be brought for us and if possible, government should make it a free service.

R5 male: Mine is a question. About keeping my sample, like we said earlier for future use, what are they keeping and is it food? Why would they need to ask me for it again?

**Mod: Thank you all for your time and participation.**

END

**FOCUS GROUP DISCUSSION 010 GRASSROOTS COMMUNITIES YOUTH, WEST NILE**

**Mod Qn.1: What are some of the things that we can inherit from our parents?**

**R1 male:** One thing one can inherit from a parent is behavior

**Mod probe:** Ok, any other person?

**R4 female:** Another one is diseases

**R5 female 2:** You can also inherit physical resemblance and skin complexion

**R4 female:** One can also inherit the same blood group as that of the parents

**R male:** Some people also inherit a strong IQ level from their parents

**Mod Qn. 2: What health conditions can we inherit from our biological parents?**

**R3 male:** Could it be the health teachings? or, the good feeding?

**Chorus:** Mild laughter

**R2 male:** Come again with the question

**Mod probe:** **What are some of the health issues that one can inherit from a parent?**

**R3 male:** I think it has to do with personal hygiene habits

**R6 female:** I would also think it has to do with one’s parents sending him or her to school to get health education.

**Mod probe:**

**Chorus:** Laughter and silent discussions.

**R2 male:** We have not understood the question very well.

**Mod probe:** **What are some of the health conditions that a child might suffer from originating from the parents?**

**R4 female:** I think the feeding a mother gives is what can bring about some health issues.

**Mod Qn3:** **What do you understand by the word gene?**

**R3 male:** Evil spirits of cause

**Chorus:** Mumbled laughter and discussions

**R2 male:** I think to help us understand this question well, what is the meaning of genes?

**Mod:** **Genes are biological traits that one can inherit from their biological parents in the event of reproduction.**

**R5 female:** From what I know, genes are things that can be transferred from the biological parents to their child, for example if one of the parents has a history of diabetes, the child may inherit that too.

**R5 female:** One can inherit the parents’ skin color

**R3 male:** One can also inherit characters of the parents.

**Mod:** What do you know about DNA?

**R2 male:** What now is called DNA?

**Chorus:** Laughter

**Mod:** **If you have ever heard of two people fighting over a child and they go to the hospital to prove whose child it is, what the hospital uses is called DNA testing. What do you know about this?**

**R3 male:** All I know is that it can be the skin complexion and appearance of the father, because you can tell some people’s parents by the resemblance.

**Mod probe:** **Any other person?**

**R4 female:** I know that for example if two people are fighting over a child and they go to the hospital, they remove something from both the father and the child to test and if it’s the actual father, what is removed from both can almost look the same, 99% similar in nature but not exactly the same.

**Mod:** Thank you, any other view?

**All:** Silence

**Mod Qn.4:** What do you know about Genome?

**R2 male:** For me I have no idea

**Chorus:** Laughter

**R5 female:** For me I think if genes are mixed together with DNA, it results in to what you are calling genomes.

**Mod probe:** **Thank you lets speak a little louder for the recording; any other idea?**

**All:** Silence

**Explanations**

***Thanks for your contributions. Let us share more about these two issues. Each species or group of organisms has a unique set of inherited characteristics that make them different from each other e.g. color of skin, eyes, height. These characteristics are usually encoded/incorporated in the DNA molecules present in their cells.***

***DNA is the genetic building blocks that govern all the characteristics of a living thing (e.g. animals, plants, and bacteria).***

***Genes are the elements of heredity that govern what is transmitted from parents to offspring in reproduction. Genes are composed of specific DNA portions or segments that are capable of controlling specific heredity characteristics like eye color, skin color height etc.***

***Genome is an organism’s complete set of DNA, including all of its genes. Each genome contains all of the information needed to build and maintain that organism.***

***An organism has one genome, but it has thousands of millions of genes in that particular organism.***

***Ethical issues***

***Your genetic information can be similar to that of your immediate family, and blood relatives. Your decisions concerning testing and what to do with the results may affect them as well.***

***Health professionals are ethically responsible to prevent harm to those involved. The individual tested also has responsibilities and obligations and should appreciate the shared nature of genetic information within families.***

***In some cases, genetic tests provide reliable and accurate information based on which decisions could be made e.g. … while in some cases, it is not possible to get definitive results. Prediction of a potential condition or disease may not include the severity of the condition or the age for the onset of symptoms. An individual is much more than the sum of their genes: the individual’s environment can modify the expression of genetic messages to the body. Many health factors are not genetic.***

# Attitudes towards applications of modern genetics and genomics

We are going to discuss specific issues to do with genetics and genomics. We would like to hear your views from the stand point of your ethnic group or culture and from your religious back ground. You could also tell us what you personally think.

## 5. Genetic testing

*A genetic test examines your DNA, and can reveal changes or variations in your genes that may be associated with an illness or a disorder. A genetic test can be arranged by your doctor or health clinic.*

**Mod 5.1:** **Who among us has experience with taking a genetic test?, (Could be you or someone close to you). What was your experience with the process of getting the test?**

**All:** Silence

**Mod:** No one?

**Chorus:** No

**Mod Qn.5.2:** **What is your opinion on bringing these test services within your communities in Uganda so that people with a family history of serious genetic diseases can find out if they are at risk?**

**R3 male:** I think it would be good to bring such services within our communities because there are common cases of people who deny their children that are conceived outside marriage.

**R6 female:** For me I think it’s necessary because there are certain diseases that need to be tested from the root cause like breast cancer or cervical cancer.

**Mod probe:** **Any other view?**

**All:** Silence

**Mod Qn.5.3: What do you think about the idea of informing people with whom you live or work the results from your genetic tests?**

**R1 male:** My view is that your test results should only be given to you because they are private and will only affect you.

**R3 male:** For me I think it’s good to tell your relative so that he can be able to explain to you in better terms.

**R5 female:** I think it’s a bad idea because people who do not like you take advantage of the information to spread bad information about you and you become the talk of the town, so I think it’s best to give it to the owner of the results.

**Return Of results:**

**Mod Qn.6.1: If there was a study on GG that could potentially reveal that you/ your family or community is susceptible to certain diseases which are very difficult to treat, would you consider participating? Would you want the research results if you participated? Would you be willing to share the results with immediate family, blood relative, and community and beyond?**

**Chorus:** Yes

**R1 male:** Yes I would be willing to go for the tests

**Mod probe: Why would you want that?**

**R1 male:** I would very much want to know if I also have such a disease in my blood

**Mod probe: Any other person with a different reason?**

**R4 female:** I would accept to know the level of the sickness so that I can also try to get a solution for my cure.

**Mod Qn.6.2: In case you agree to carry out these genetic tests, what are some of the things that you think can convince you to comfortably take the tests?**

**R6 female:** I think I need guidance and counseling, otherwise I might get shocked by the findings.

**Mod probe:** Any other view?

**Chorus:** Quiet discussions.

**Mod probe:** **The main issues here are as we have said, your samples will be preserved for future studies, your results will be kept secret or exposed to your people and your samples will be used for helping others in your family, country or internationally. With this knowledge, what would help you comfortable enough to take the test without hesitation or doubt or fear?**

**R5 female:** Knowing that my results will be kept a secret gives me peace, ‘laughs’.

**R6 female:** For me the fact that it will be used away from my home in another country gives me peace, in fact it should only be used outside the country, may be only my closest family should know.

**R4 male:** I will willingly accept the tests because I know it will help my family in the near future.

**Mod Qn 6.3:** **Would you still accept to take the test if it was for testing a suspected hereditary disease?**

**R1 male:** Yes I would accept

**R5 female:** Yes it is a good reason to accept because if there is a serious hereditary disease, it can be detected early enough to help the rest of the family generations.

**Mod:** **How would you prefer the results of your genetic tests to be revealed to you?**

**R4 female:** Whoever is breaking the new has to seat calmly face to face with me to explain the results for me to understand well, without going in to a shock.

**Chorus:** Laughter and chorus discussions.

**R6 female:** For me I would need to be counseled properly before giving me the results.

**R5 female:** For me I want to be with my parents when I am getting the results from my hereditary testing.

**Mod probe:** **Any other?**

**All:** Silence

**Mod**: **Who should be given your hereditary testing results, you or your family members?**

**R4 female:** Both parties should know

**R6 female:** Both should know

**R3 male:** The result should be given to both you and the one who will take care of you so that he/she can understand what you are going through and be there to console you.

**Mod probe:** **How do you think the result should be delivered to you?**

**R1 male:** I think the care taker should be privately called aside to be given the result and he/she should keep it away from the patient.

**Mod:** **After getting such result, would you be willing to share it with your close family, relatives and community?**

**R3 male:** For me I would tell all of them, so that they are aware of what the doctor has advised me to do and stand with me in support.

**R2 male:** It is good for the community to know because these days people assume someone’s death up to the extent of accusing other community members with whom the person could have had a grudge, in order to avoid such assumptions, they should know.

**R5 female:** The family members need to know because some diseases may need extra attention and care like meals on time, special foods etc, so that the family members can be helpful in looking after you.

**Mod Qn.6.4:** **During genetic testing, other issues which were not of focus may also be found, should such extra results be revealed or kept silent? Why?**

**R4 female:** I don’t want those revealed because some people don’t know how to keep quiet about such news.

**R2 male:** They should tell me everything so that I know the state of my health.

**R5 female:** The initial finding can be revealed to me and my family jointly but the extra findings should only be told to me to keep to myself.

**All:** Laughter

**R6 male:** I want them to tell me everything whether the original disease suspected was found or not, other issues found, everything in the exact state found, so that I know.

**R2 male:** I also want to know all the diseases found in my body because each of them will need different medication, so that I will tackle the ones I can for saving my life.

**Mod:** **The findings from hereditary testing are the genetic information, should this information be revealed to other community members? How should it be conveyed?**

**R4 female:** This information should only be revealed to my family and they should be advised how to handle the information but not to other community members because some people may start isolating and talking about you when they know what you are suffering from, which may cause stigma.

**R5 female:** For me other people can be told so that everyone is aware of what I am suffering from and if they are needed to help, they will come willingly.

**R6 female:** Other people should be told so that they are aware of the kind of diseases amidst us and learn to take care of themselves better.

**R3 male:**’ Laughs’ for me I think only my family members should know such results because I know they will understand and be mindful of how to handle it unlike outsiders who will not care about your feelings and go around talking recklessly.

**Mod Qn:** **Is there any other thing about hereditary testing that we have not mentioned but you would like to share with us?**

**R4 female:** No, for me I don’t have anything else to say about it.

**Mod probe:** **Any other person, how about you?**

**R1 male:** There is nothing more to say, we should just end.

**All:** Light laughter

**Mod:** **Ok, if we all have nothing else to say, that ends our discussion, thank you for your time and contributions.**

**END**
