# Supplementary material for: Feedback of individual genetic and genomics research results: A qualitative study involving grassroots communities in Uganda
Source: PLoS One. 2022 Nov 18;17(11):e0267375. doi: 10.1371/journal.pone.0267375 (PMC9674126; doi:10.1371/journal.pone.0267375)
Supplement: Attachment — Submitted filename: pone.0267375.s002.DOC [file pone.0267375.s002.DOC]

The Editor,

PLOS ONE

Dear Sir/Madam

Re: Feedback of Individual Genetic and Genomics Research Results: A Qualitative Study Involving Grassroots Communities in Uganda [PONE-D-22-10220] - [EMID:b03a6789c1bfee68]

**Point by point response to review comments**:

Following is the point-by-point response to review comments.

Please include the following items when submitting your revised manuscript:

- A rebuttal letter that responds to each point raised by the academic editor and reviewer(s). You should upload this letter as a separate file labeled 'Response to Reviewers'.

**Response: This has been done**

- A marked-up copy of your manuscript that highlights changes made to the original version. You should upload this as a separate file labeled 'Revised Manuscript with Track Changes'.

**Response: This has been done**

- An unmarked version of your revised paper without tracked changes. You should upload this as a separate file labeled 'Manuscript'.

**Response: This has been attached.**

If you would like to make changes to your financial disclosure, please include your updated statement in your cover letter. Guidelines for resubmitting your figure files are available below the reviewer comments at the end of this letter.

**Response: No**

Journal Requirements:

When submitting your revision, we need you to address these additional requirements.

1. Please ensure that your manuscript meets PLOS ONE's style requirements, including those for file naming. The PLOS ONE style templates can be found at

<https://journals.plos.org/plosone/s/file?id=wjVg/PLOSOne_formatting_sample_main_body.pdf>  and

<https://journals.plos.org/plosone/s/file?id=ba62/PLOSOne_formatting_sample_title_authors_affiliations.pdf>

**Response: This has been done**

2. In the ethics statement in the Methods and online submission information, please ensure that you have specified what type you obtained (for instance, written or verbal, and if verbal, how it was documented and witnessed). If your study included minors, state whether you obtained consent from parents or guardians. If the need for consent was waived by the ethics committee, please include this information.

**Response: Information stating that “individuals of 18 years and above who had provided written informed consent participated in the study”**

**We have also added information that “For non-literate participants, the consent process was witnessed by a literate individual who was independent from the study and the written consent was documented by a thumb print of the participant followed by signature of the witness”.**

3. Thank you for stating the following financial disclosure:

“Research reported in this publication was supported by the National Human Genome Research Institute of the National Institutes of Health under Award Number U01HG009822 and NIH Fogarty grant under Award Number D43TW01511. The content is solely the responsibility of the authors and does not necessarily represent the official views of the National Institutes of Health.”

Please state what role the funders took in the study.  If the funders had no role, please state: "The funders had no role in study design, data collection and analysis, decision to publish, or preparation of the manuscript."

If this statement is not correct you must amend it as needed.

Please include this amended Role of Funder statement in your cover letter; we will change the online submission form on your behalf.

**Response: The funder had no role in the data collection and analysis, decision to publish or preparation of the manuscript.**

4. We note that you have stated that you will provide repository information for your data at acceptance. Should your manuscript be accepted for publication, we will hold it until you provide the relevant accession numbers or DOIs necessary to access your data. If you wish to make changes to your Data Availability statement, please describe these changes in your cover letter and we will update your Data Availability statement to reflect the information you provide.

**Response: Thank you. This is noted and no changes are planned.**

[Note: HTML markup is below. Please do not edit.]

Reviewers' comments:

Reviewer's Responses to Questions

**Comments to the Author**

1. Is the manuscript technically sound, and do the data support the conclusions?

The manuscript must describe a technically sound piece of scientific research with data that supports the conclusions. Experiments must have been conducted rigorously, with appropriate controls, replication, and sample sizes. The conclusions must be drawn appropriately based on the data presented.

Reviewer #1: Yes

Reviewer #2: Partly

Reviewer #3: Yes

2. Has the statistical analysis been performed appropriately and rigorously?

Reviewer #1: N/A

Reviewer #2: I Don't Know

Reviewer #3: N/A

3. Have the authors made all data underlying the findings in their manuscript fully available?
666666666
The [PLOS Data policy](http://www.plosone.org/static/policies.action#sharing) requires authors to make all data underlying the findings described in their manuscript fully available without restriction, with rare exception (please refer to the Data Availability Statement in the manuscript PDF file). The data should be provided as part of the manuscript or its supporting information, or deposited to a public repository. For example, in addition to summary statistics, the data points behind means, medians and variance measures should be available. If there are restrictions on publicly sharing data—e.g. participant privacy or use of data from a third party—those must be specified.

Reviewer #1: Yes

Reviewer #2: Yes

Reviewer #3: No

4. Is the manuscript presented in an intelligible fashion and written in standard English?

PLOS ONE does not copyedit accepted manuscripts, so the language in submitted articles must be clear, correct, and unambiguous. Any typographical or grammatical errors should be corrected at revision, so please note any specific errors here.

Reviewer #1: Yes

Reviewer #2: Yes

Reviewer #3: Yes

5. Review Comments to the Author

Please use the space provided to explain your answers to the questions above. You may also include additional comments for the author, including concerns about dual publication, research ethics, or publication ethics. (Please upload your review as an attachment if it exceeds 20,000 characters)

**Reviewer #1:** This manuscript reports on the results of deliberative focus groups conducted with members of grassroots communities in Uganda. These focus groups explored views on return of results from hypothetical genomic research. The findings are interesting and important due to the paucity of research on this topic in non-USA populations. As a general comment, the manuscript would benefit from proofreading for punctuation. I have the following specific comments:

Abstract
Line 58. Please specify which type of content analysis was employed (ie., deductive versus inductive).

**Response: Thank you for this opportunity to clarify. Information has been added to the abstract to indicate that thematic analysis was conducted both deductively based on the predetermined study questions and inductively for identification of any emerging themes. Line 65-68 tracked copy**

Line 69. Full stop missing.

**Response: We apologize for this error. A full stop has been added. Line 83 tracked copy**

Line 70. Use GGE rather than writing in full.

**Response: This has been done. GGR has been used to replace genetics and genomics research. Line 83 tracked copy**

Line 72. The authors state “The strong therapeutic misconception linked to GGR is concerning…” but this doesn’t make sense unless the results within the abstract mention that the hopes for benefit are unfounded.

**Response: This has been improved with a statement “findings suggested strong therapeutic misconception linked to GGR is which is concerning** **given that hopes for diagnostic benefit are unfounded…” . Line 85-87 tracked copy**

Introduction
81. Really long sentence. Consider splitting up. It would also be good to explain these challenges in a little more detail.

**Response: The opening sentence of the introduction has been modified to make it clearer.**

**The challenges associated with GGR are listed and explained in the second sentence of the introduction as well as subsequent sentences. Line 99-106 tracked copy**

94. Consider deleting “with resultant literature” as it is implied.

**Response: With resultant literature has been deleted. Line 113 tracked copy**

95. Another really long and hard to follow sentence.

**Response: The sentence has been split and modified. Line 112-123 tracked copy**

104. The authors state debate is limited but then list 7 studies. It would be good to highlight what exactly about these papers is lacking to justify the need for the current study.

**Response: Thank you for this comment. However, we would argue that only seven studies in the whole of Africa is quite low give the diversity of African populations. For Uganda, especially, there is much less literature to inform context specific ethical guidelines. Additionally, debate particularly concerning the grassroots communities is lacking or limited.**

113. It would be good to define grassroots communities.

**Response: Thank you for this comment. We have defined grassroot communities towards the end of the introduction as “grassroots communities who are populations based in rural settings and are considered to have limited interaction with the outside world,** **less formally educated and represent the lower socio-economic groups”. Line 142-145 tracked copy**

Methods
128. I think the authors mean focus groups, not interviews.

**Response: Thank you for pointing out this error. This has been rectified as FGDs. Line 164-165 tracked copy**

131. The information about the number of focus groups conducted should be in the results section.

**Response: The number of FGDs has been moved to be the first sentence of the results section. Line 233-239tracked copy**

139. Please provide more information about how participants were recruited. How were the selected? Who approached them and how?

**Response: Information has been added to the methods section as “The research assistants worked with a local community mobilizer to identify the potential participants and invited them to a meeting with the study team”. Line 172-180 tracked copy**

161. Please provide a reference for the type of analysis you used.

**Response: References number 29 and 30 have been added. Line 212 tracked copy**

Results
It’s great that the authors have listed which FG the participant quotes are from but it would also be good if each participant in each FG was numbered as well. This adds rigour to the results because it shows it’s not the same person speaking all the time, eg. lines 358 -366.

**Response:** **This is noted and has been addressed throughout the results section. It is indicated in the text as well.**

189. Content analysis identifies content categories, rather than themes.

**Response:; We conducted thematic analysis.**

**This has been clarified and more information added as; ‘‘The coding was done both deductively based on the study predetermined themes and, inductively to identify emerging themes.’’ Line 206-212 tracked copy**

215. Can the authors please clarify whether positive and negative here mean gene-positive/negative or positive/negative from the point of view of the participant? Not currently clear.

**Response: This meant the results being positive with the genes for a particular genetic predisposition under investigation. And negative is when the gene under investigation is not identified in an individual.**

446. This quote was already used in the genetic counselling section. Not great form to reuse quotes.

**Response: The quote has been deleted from the section of privacy and confidentiality. Line 571-572 tracked copy**

448. Use of the word ‘noise’ doesn’t make sense to me. Maybe check translation?

**Response: This has been improved to read as loud noise which can trigger some epileptic attacks. Line 613 tracked copy**

Discussion
557. Are these suggestions for addressing fears based on your data or other literature?

**Response: This was line 577 in the original submission. The suggestion for addressing fears is based on our data and is in all sections; for example, the section on implications, informed consent and genetic counselling, respondents stress the need for adequate preparation of the individual before testing and before feedback of findings aimed at mitigating the negative effects.**

590. Again, I don’t think you really made this point about the therapeutic misconception explicit in the results. Make it clearer so we know what you are referring to when you discuss it in the results.
Consider breaking up some of the long paragraphs into separate ones for ease of reading.

**Response: –The results have been revised to include therapeutic misconception a sub-theme 1.1 for theme one. Line 234-264 of the tracked copy.**

**In the discussion has been improved to highlight therapeutic misconception. Line 741-753 tracked copy.**

681. Is this true? Australian laws allow this but I am not sure about Belgium so I would check this. There’s a paper by Phillips et al from last year that specifically discusses Belgian Laws on this issue.
It would be good if the findings of this study were contrasted more with others, particularly from rural communities.

**Response: This has been rectified. It is the Australians, Britain and French that have legislations, while *c*urrent Belgian legislation coupled, with international precedent, may provide sufﬁcient justiﬁcation to establish a duty to inform relatives of their genetic risk in some cases. Discussion has also highlighted the fact that applicability of the western concept of privacy in the Uganda setting may need reflection. Line 821-827 and 861-867 tracked copy**

**Reviewer #2:** The aim of this paper is to assess the perceptions of grassroots communities in Uganda regarding the feedback of genetic and genomic results to patients/participants, presumably with the goal of helping to inform guidelines and or policy regarding the feedback of individual genetic/genomic results in this context. I have a few questions regarding the author’s interpretation of their results and statement of their conclusions, which would need to be addressed prior to the paper being suitable for publication.
1) The primary issue is with the author’s claim that, according to their results, the feedback of genetic/genomic results “was acceptable to all respondents” (Line 566). First, it would be worth being explicit that this acceptance comes with certain conditions, which the authors describe in the body of the paper (e.g., informed consent, the condition being treatable). More importantly, however, it is not clear that what the feedback of research results would involve in practice is relevantly similar to what the participants in this study envision, and thus, I’m not sure that the authors can really say that these participants would find the feedback of genetic/genomic results ‘acceptable.’ The authors tacitly acknowledge this when they raise the issue of the therapeutic misconception, and point out that most genetic studies may not yield results that can benefit the participants. Yet this seems to be a pervasive assumption on the part of the participants. I think the authors need to be clearer that the hypothetical situation the participants seem to take themselves to be responding to may not correspond to the reality of feedback, and this should be borne in mind when extrapolating on the basis of their study findings.

**Response: Information has been added to the opening section of results to read; However, the willingness to participate in GGR was based on the condition for feedback of results. Other expectations included but were not limited to adequate informed consent, genetic counselling, implication, privacy and confidentiality.**

**Additionally, its indicated that this feedback is in the context of genetics and genomics research. Line 243-246 tracked copy**

**It should be noted that, the hypothetical situation the participants seem to take themselves to be responding to may not correspond to the reality of feedback, and this should be borne in mind when extrapolating on the basis of their study findings. ”**

**Most participants came up with hypothetical situations. If they received results in reality, it might be very different. Line 736-739 tracked copy**

2) Similarly, it would be useful if the authors could be a bit more specific about the context in which feedback is occurring. Is it research involving genetic/genomic testing? Genetic/genomic testing as a part of clinical care? Are the results only individual-level research results, or might this include secondary or incidental findings?

**Response: The context in which feedback of results is discussed is research involving genetics/genomic testing. A clarification has been added to the opening section of the results section.**

**The respondents were agreeable to receiving all genetic/genomic research results including individual, incidental and secondary findings. However, for this paper, we have focused on individual results. This information has been added to the introductory section of the results. Line 255-258**

**Although participant willing to receive results was based on their mistaken belief of health benefit - therapeutic misconception. . The discussion section has also been edited - lines 736-739. …….acceptable to all respondents conditional upon receiving health benefits.**

A few other small points:

3) On Line 87, the authors mention ownership of DNA; does this mean ownership of the biological sample, or the data derived from sequencing the genome?

**Response: It’s the ownership of both the sample and the associated data. This sentence has been revised. Line 105-106 tracked copy**

4) On Line 101, the authors state that there is consensus that several conditions must be met before feedback of results is permissible, including “ensuring that patients are appropriately informed of the implications of the findings.” If the question is whether feedback should happen or not, how can informing the patient of the implications be a deciding factor (in order to inform them of the implications, wouldn’t we needed to have informed them already?)

**Response: yes, the question was whether feedback should happen, and if so, how should be appropriately be carried out. This addresses some of the possible negative effects of such feedback.**

5) I wasn’t sure what the authors meant when they stated that coding was done ‘both inductively and deductively’. A bit more description of how new codes were generated, and their justification, would also be useful.

**Response: Information has been added as; coding was done deductively based on the study predetermined themes and, inductively to identify emerging themes. Line 206-212 tracked copy**

6) It wasn’t clear to me exactly why ‘grassroots communities’ in particular were an important population to engage with on this issue; is there a reason to think that their views would be different than participants in other contexts. More generally, a few sentences early on describing why the numerous ethical guidelines for feeding back results might not be applicable to the Ugandan context would really help to motivate the paper. The section beginning on Line 647 is a really nice example of why policies developed based on other socio-cultural contexts might not be transferable to the Ugandan context, and it would be good to highlight this as one of the important

**Response: Thank you for the comment.**

**The purpose for this study was to generate data that would inform context specific guidelines. Hence the need to include as many stakeholders as possible. Other stakeholders like researchers, research participants, REC, patients and regulators were studied (references 26, 27, 28) but not the subject of this manuscript.**

**Information has been added to the introduction section as: Addition of the views of the grassroots communities to those of other stakeholders in research will provide a more comprehensive context regarding guideline development and conduct of GGR in Uganda. Line 145-148 tracked copy**

**Reviewer #3:** This paper reports the findings of a series of deliberative focus group discussions exploring community attitudes to the feedback of genomic research results. It provides a valuable addition to the literature by reporting on Ugandan community attitudes, a region relatively new to genomic research and with whom little research of this nature (ELSI) has been undertaken. The findings have the potential to usefully inform guideline and policy development in this region.

However, there were some problems with the manuscript that require attention prior to publication.

Major:
* 'research results' needs to be clearly defined early on. It's really not clear what you mean by this (overall research findings? individual health information? raw genomic data or interpreted info? just actionable results, or any findings of interest?) and this has implications for interpreting the results. There is no reason why individual health info should be shared or would be shared with the whole community, but it is widely expected that research findings will be communicated with the wider participating community, especially if there are potential ramifications for socially identifiable groups. It's also not clear whether this was adequately explained to the focus groups in the education component.

**Response: Thank you for the comment.**

**During data collection, there was an education session lasting about 30 minutes where genetics and genomics was explained to the participants including participation in GGR. And the participants were aware and understood that they were discussing aspect of GGR during the FGDs. Information has been added to the methods section to indicate what was explained in the educational session. Line 192-197 tracked copy**

**It is also true that findings from our study reveal that the participants at the grassroots communities want to know all the GGR results whether actionable/beneficial or not. The participants were very clear that if anyone is tested, them they expect the test results.**

**Participants also felt that any genetic predisposition may affect their life either immediately or in the future, hence the need to learn such information and plan for their future as well as their loved ones. Participants expressed willingness to know the results of their genetic tests both as individual results, incidental findings and also secondary findings. Line 255-258 tracked copy**

**However, for this manuscript were focused on individual results.**

**Additionally, most of the respondents are willing to share their GGR results with their close ones for reasons like social, economic and psychological support. It should be noted that in our Ugandan setting, family is always crucial in supporting their loved ones in case of any health problems including meeting the cost of treatment as well as providing nursing care.**

**For individual health information, it is the information generated from one’s participation in GGR that can affect one’s health. Because a test can generate results that reveals presence or absence of particular traits, and this in turn has implications for the health of an individual.**

* In the introduction, you need to briefly outline the findings of previous research, rather than just say that 'There have been a few publications on perspectives...' - this will give readers an idea of what to expect, and is also an opportunity to highlight why we might expect Uganda/grassroots communities to differ from other cohorts.

**Response: Information has been added as follows: Available local literature which has highlighted the need for adequate informed consent, community engagement, genetic counselling, feedback of beneficial/actionable GGR findings is what is highlighted by the researchers. But research participants added a component of more effective support to research participants during and after participation as well as feedback of all findings since consideration of beneficial/actionable findings to a good extent is individual based. Line 134-141 tracked copy**

* on what basis were the communities selected?

**Response: The communities were selected on the basis of being rural and removed from the urban settings, with less outside influence, relatively low socio-economic status, less educated and representing a major ethnic grouping in the country. In this case it was the Luo, Nile hermites and Nilotics. Line 172-180 tracked copy**

* what language were focus groups held in? transcripts? were you interpreting as you went, or were discussions translated later? were back translations employed for accuracy checks?

**Response: The FGDs were conducted in the respective local languages spoken by the members of the community for each of the ethnic groups. Data was transcribed verbatim and later translated to English, as indicated in the methods section. Sub-sections on data collection and data management. Line 188-190 and 204-205 tracked copy**

* what do you mean by 'a safe distance' was this about Covid or people overhearing?

**Response: The safe distance away from non-participants was to ensure privacy and to abide by Covid-19 protocols. This has been added to the sentence. Line 183 tracked copy**

**Concerning COVID-19 prevention, it is indicated in the next sentence that the COVID-19 mitigation plans like social distancing, face masking and hand sanitization were employed. Line 184-186 tracked copy**

* the qualitative analysis sounds more like a form of thematic analysis than content analysis? the methods section would be strengthened by references to relevant theoretical/methodological literature and greater detail on what was done

**Response: This has been revised to clearly indicate thematic analysis. Reference number 29 and 30 to support the analysis write up. Line 212 tracked copy**

* how well was the research context and how it differs from the clinical context discussed/understood? there is significant evidence of therapeutic misconception throughout - some of this just be how people understand research, but some may have arisen through lack of clarity from the team here, eg the participant refers to themselves as 'the patient', they all want to know their 'health status' and assume they are sick or the researchers will find a disease

**Response: The participants were not sick and were not in a clinical setting. All were invited from their homes to come for a meeting to exchange views on participation in research.**

**They were clear when they said that if you test for anything, you need to know the test results.**

**They were also clear when they mentioned that when you test, you may find either positive or negative findings. This means that they may get findings that reveal a particular trait or not, and this can impact negatively on their health. The findings of such testing would help them plan their future.**

**However, wanting to know one’s health condition in a research setting may constitute therapeutic/diagnostic misconception. This is highlighted where participants felt that sometimes GGR could reveal information that impacts their health either immediately or in the future. Hence knowing the results would help them get treatment where it is available. This has been discussed. Line 741-753 tracked copy**

* actually, 'therapeutic misconception' would be a useful thematic section in the results

**Response: Yes, we acknowledge that therapeutic misconception can be a theme in our results. We have revised the results section to create a sub-theme 1.1 under theme one, of the need to know one’s health. Line 324-362 tracked copy**

* the results section requires significant reworking - there are numerous sections where the illustrative quotations don't relate to the point just made, and other sections where points are out of order. The quotations chosen have a lot of potential to be illustrative of interesting and useful points, but are currently not well integrated.

**Response: The results section has been improved and a mis-aligned quotation removed as well as adjustment of quotes. Line 401-405 tracked copy**

* in a lot of places, phrases like 'participants reported that...' or 'respondents felt that...' need to be inserted, to avoid statements like 'researchers are obligated to provide such feedback' - which are not established by the evidence here. Similarly, where you say 'including the fact that it helps individuals to know what to do in their life' - this isn't a fact, it is a perception. 'It can guide the medical professionals and scientists to search for treatment' - it MAY do this, but it depends on a lot of factors. Language is too strident in places.

**Response: The write up has been revised and the issues have been addressed using appropriate language.**

* need to define 'ancestral information' - unclear if you are referring to maternity/paternity, inheritance patterns, population genomics or something else. There was a lot of focus on (non) paternity, which is a risk in some research projects but certainly not all, and this distinction was not made clear. Similarly, there seemed to be an assumption that everyone would have some health information come out of genomic research participation, which is a long way from the truth.

**Response: Thank you for this comment;**

**Ancestral information is actually the Ancestry testing for inheritance patterns. Line 354-355 tracked copy**

**Although paternity testing was not the basis for this study, it was used to explain the concept of genomics because it is well understood by the general population, the focus of this paper is return of individual genetics and genomic research results.**

**Yes, genetic and genomics research and information generated does impact health of an individual. If the findings reveal no particular gene that causes genetic predisposition to a particular disorder, one considers themselves normal concerning that disorder. If the findings reveal a genetic predisposition to a particular disorder, then it negatively impacts the health of the individual tested.**

**Additionally, in our discussion, we tackled the issue of therapeutic/diagnostic mis-conception where individuals might have mistakenly felt that all GGR comes up with some form of results. Line 741-753 tracked copy**

* the section where participants were described as concerned about the cost of genomic research was unclear - research participants would not normally bear these costs, nor should participation be seen as desirable for access to genomic testing (potentially undermining free consent and giving a deceptive view of the information/benefit to be gained from participation). The kind of feedback the participants seemed to envisage is very much not what most studies would provide - the finding that this is an expectation could be very useful for designing ethical guidelines, but this is undermined a bit if actually the participants just weren't given accurate information to begin with.

**Response: The participants were taken through an educational session and given information in the form of research scenarios that would reveal information about someone’s genetics and genomics traits. They were agreeable to research participation but wondered if such genetic testing services would be available and affordable to the research community post the research period. line 192-197 tracked copy**

**This raises the issue of community benefit that would normally arise if the research conducted was responsive to community health needs and the intervention proved to be efficacious/ implemented.**

**This is more of highlighting the need for community benefit following participation in research rather than not understanding the research.**

**It is listed as a challenge where participants felt that following research, the uptake of the research into practice may not benefit the poor individuals because of issues of affordability.**

**We have added to the discussion to include aspects like meaningful engagement of communities where research is conducted to create collaborative partnerships between researchers and communities with resultant capacity building, technology transfer and benefit sharing aspects. Line 839-844 and 884-905 tracked copy**

* privacy and confidentiality discussion would be strengthened if discussed within the context of legal obligations in this area - esp in the discussion, where it's not clear whether the problems identified with confidentiality are easily accommodated within existing laws/regulatory frameworks or not

**Response: Thank you for this comment.**

**The discussion on privacy and confidentiality have been improved to highlight available legislation and guidelines in the Ugandan context. Line 821-827 and 854-869 tracked copy**

* the variability in attitudes to feedback (to individuals/families/wider community) indicate that working out preferences for this should be part of the consent process

**Response: Thank you. This is implied in the discussion where it is stated that informed consent will be obtained before testing and will involve aspects like return of results. And for those who choose to receive feedback of results, they should again be taken through both informed consent and genetic counseling at the time of feedback of results. Hence the consent will be done at different levels and based on individual’s acceptability to receive feedback of results. More Information has been added to the discussion; And for feedback of GGR findings, consent should be sought at different levels including before recruitment of the participant, and at return of results for those who chose to received results. Line 760-763 tracked copy**

* the discussion is also light on - there is a lot in the results that could usefully be contextualised, but the discussion needs significant revision (unclear what you mean by 'this type of feedback of results', 'the need to know one's health condition' etc) - the strategies suggested were a bit meaningless (complying with ethical and legal requirements like privacy is not really a 'strategy') and didn't make the most of the really interesting data you've collected

**Response: The discussion has been revised to address the comments including discussion of available legislation on privacy and detailed discussion of the challenges like genetic vocabulary, community engagement, community benefit, capacity building and how they can be addressed. Line 821-827 and 839-844 and 884-905 tracked copy**

* 'revealed recall bias about their participation in the concerned research study' - what do you mean by this?

**Response: The study was meant to capture experiences of individuals who had participated in a genomics study. However, by the time the evaluation study was done, many of the respondents did not have an accurate recollection of the informed consent experiences in the genomics study. Hence, respondents answered based on what they could recall. Line 765-767 tracked copy**

* there are important implications for how we conceive of researcher obligations, benefit sharing, and capacity building priorities, but these are inadequately drawn out here

**Response: Information has been added to the discussion to capture this comment. Line 839-844 and 884-905 tracked copy**

* would be good to see community research agreements discussed as part of the community engagement discussion

**Response: Thank you for this comment. We stressed the need for meaningful community engagement and if achieved would yield fair collaborative arrangement between researchers and the community where the research is conducted for mutual benefit. Line 839-844 and 884-905 tracked copy**

* acknowledgement of the familial implications (and even for ethnic groups/communities) of genomics, in addition to caring responsibilities as a reason to discuss findings with the community (not individual health data though! more general research findings)

**Response: The discussion has been revised to capture the comments. Line 884-905**

* if the point of this project is to inform guideline development, this should be mentioned in the introduction, not the closing paragraph.

**Response: This has been done earlier in the introduction. Line 147-148 tracked copy**

* the limitations mentioned are extremely significant. Adding some comment on how these were managed/mitigated or how they impacted (or didn't) on the results would be useful.

**Response: The limitation section has been revised to add information on how the limitations were mitigated.**

* the discussion needs to analyse and interpret the findings with more nuance/attention to context/thoughtfulness to be useful, given the extent of the literature in this area. Closing with 'Privacy and confidentiality, benefits, risks as well as implications for sharing need to be considered...' adds nothing to the literature and wastes the data you've collected.

**Response: The discussion has been revised to capture the comments.**

Minor:
* Too many novel acronyms, none of which will be used in another paper and which only serve to distract the reader. Just write in full.

**Response: We have removed the UNCST. But the other acronyms have been retained because the phrases appear several times.**

* Ownership of DNA - do you mean the data? or the actual sample? bit odd to refer to the DNA rather than the data or sample.

**Response: It is ownership of the sample and associated data. This has been clarified in the write up. Line 47 tracked copy**

* some awkward phrasing throughout - revise for clarity

**Response: The write up has been revised and improved.**

* 'They were assisted by eight research assistants including four females proficient in local languages.' Females should be women, and this sentence is ambiguous - are you saying the four men were not proficient in local languages?

**Response: For considerations regarding gender sensitivity, both men and women fluent in the respective local languages were employed as research assistants. Line 165-167 tracked copy**

* not sure 18-35 can be described as youth - 'younger participants' maybe?

**Response: In Uganda, the youth are younger individuals aged 18-35. Line 170 tracked copy**

* 'if one has a 50% chance of developing' - why 50%? do you just mean if risk is quantifiable?

**Response: It is a probability of developing a condition for individuals who have a genetic predisposition.**

* contractions (eg it's) should be eliminated

**Response: This has been done.**

* paragraphs in the discussion are way too long

**Response: Paragraphs have been shortened.**

* legislated laws is a tautology

**Response:	This has been replaced with “legislation”. Line 862 tracked copy**

* 'Since individuals in the communities are acceptable...' this sentence makes no sense - also it is not clear why it is up to the regulators to sort it out - are current regulations inadequate? this has not been mentioned, let alone established.

**Response: This has been clarified. Yes, the country does not have ethical guidelines for GGR, and the data from this and related research will inform development of context specific guidelines.**

**So, in order to develop guidelines, the regulators are urged to use the considerations from this study data as a basis for guideline development. In Uganda, it’s the regulators who are mandated to develop guidelines and provide oversight for ethical conduct of research. Line 880-183 tracked copy**

**Thank you. I hope the responses are adequate to address the review comments.**

Sincerely,

Dr. Joseph Ochieng
