# Supplementary material for: Feedback of individual genetic and genomics research results: A qualitative study involving grassroots communities in Uganda
Source: PLoS One. 2022 Nov 18;17(11):e0267375. doi: 10.1371/journal.pone.0267375 (PMC9674126; doi:10.1371/journal.pone.0267375)
Supplement: Attachment — Submitted filename: Response to Review Comments September 25, 2022.docx [file pone.0267375.s003.docx]

September 23, 2022

The Editor,

PLOS ONE

PONE-D-22-10220R1 Feedback of Individual Genetic and Genomics Research Results: A Qualitative Study Involving Grassroots Communities in Uganda

**Point by point response to review comments**

6. Review Comments to the Author

Reviewer #1: (No Response)

Reviewer #3: This is a much improved version, but some issues remain. In the clean version, there are still instances where the data analysis is described as 'content analysis' (eg in the abstract), but elsewhere it is claimed to be thematic analysis. The method used here would be more convincing if the authors cited some literature relevant to the method they actually used.

**Response: Thank you very much for your review and comments. Our search for the content analysis in the revised manuscript has yielded no results. The methodology and abstract sections have described thematic analysis which has also been references with references 29 and 30, line 182 of the tracked copy.**

Expression is clearer throughout, but there are still errors remaining - a thorough proofread/edit would still be valuable.

**Response: The manuscript has been revised to improve on the writing throughout the write up.**

Where the issue of sharing results with family members and the wider community is introduced, it would be helpful to explain why this might be done (to be honest, I cannot think of a single example of individual genomic results being given to the wider community by the research team, nor why this would ever be done - on the other hand, communicating general study findings [not individual results] to the participating community is very common and often expected - but this distinction is not raised here).

**Response: Information on the reasons for extending feedback of GGR results to family and community has been added as;**

**Many respondents expressed the wish to share their genetic results with some family members but this can only be done after adequate consent and genetic counselling to evaluate the possible implications of such sharing. Many families in Uganda share health and medical information because to a great extent the family members meet the cost of treatment. Sharing genetic results is not exception particularly if the genetic predisposition has health implications. Additionally, genetic predisposition can affect other members of the family, and such sharing would act both as a warning and a preventive measure. It would also facilitate future planning for the concerned family. Line 492-500 tracked copy.**

**Additionally, sharing genetic information with members of the community would require though evaluation of the implication of such sharing. It should be noted that for those individuals who felt such sharing can be done, they proposed that is be done on a case-by-case basis and depending on the health condition identified. Hence the saying, if the genetic predisposition can make me get lost, then the community can get to know my problem and come to my rescue if I get lost. The same was for example of getting an epileptic attack where the those around may come for the rescue.**

**Individuals in the Ugandan rural settings live as communities, often sharing health information and assisting in care of others including contributions to the treatment costs of those in need. If genetics results point to a particular predisposition that may affect the health of an individual, their family or community, such information may be shared by the individual tested at their discretion with clear appreciation of the implications. Line 543-548 tracked copy.**

In the discussion, you allude to sharing of results with family and the community as being relevant when others may also be at risk of carrying the variant of concern - this needs to be made clearer, as well as noting that this type of 'sharing' does not necessarily entail revealing individual personal information (it's more in the nature of general study findings). Clearly defining different types of 'results' would have helped with this confusion.

**Response: Thank you. We have added the information as;**

#### **Sharing of genetics results with family and community was assumed to have a potential to benefit the individual tested in terms of social and financial support in case the individual became unwell, but would also act as early warning to others at risk. Extending feedback should depend on the willingness of the individual tested, nature of the genetic condition, adequate genetic counselling and appreciation of the implications and, if there is likelihood of the tested individual to benefit from such disclosure. Line 762-768 tracked copy.**

**If genetic research results point to a particular predisposition that may affect the health of an individual, their family or community, extending feedback to family and community may be done at the discretion of the individual tested based on a clear appreciation of the implications. And such sharing may involve general information not specific personal information. Line 772-776 tracked copy.**

The paragraphs in the discussion are still really long.

**Response: The paragraphs in the discussion have been edited and shortened.**

You raise the Data Protection and Privacy Act of 2019 and describe it as adopting the western approach without explaining what that is - this section needs a bit more clarity

**Response: information has been added as; The Act employs the western approach that focuses on individual privacy and not the family as a whole. Line 731-732 tracked copy**

I hope the responses are satisfactory.

Sincerely,

Dr. Joseph Ochieng
